# Supplementary material for: Volcanic-associated ecosystems of the Mediterranean Sea: a systematic map and an interactive tool to support their conservation
Source: PeerJ. 2023 Mar 29;11:e15162. doi: 10.7717/peerj.15162 (PMC10066691; doi:10.7717/peerj.15162)
Supplement: Supplemental Information 1 — A checklist and meta-data information standards for Systematic Map reports. Author’s responses provided the information reported during the preparation of the Systematic Map. [file peerj-11-15162-s001.docx]

**Table S1:**

**RepOrting standards for Systematic Evidence Syntheses (ROSES) for Systematic Map Reports.**

The ROSES form contains a checklist and meta-data information standards for Systematic Map reports. Author’s responses provided the information reported during the preparation of the Systematic Map.

| **Section/sub-section** | **Topic** | **Checklist**  **meta-data** | **Author response** |
| --- | --- | --- | --- |
| **Title** | Title | Meta-data | Volcanic-Associated Ecosystems of the Mediterranean Sea: a Systematic Map and an Interactive Tool to support their conservation |
| **Type of review** | Type of review | Meta-data | Systematic Map |
| **Authors' contacts** | Authors' contacts | Checklist | Yes |
| **Abstract** | Structured summary | Checklist | Yes |
| **Background** | Background | Checklist | Yes |
| **Stakeholder engagement** | Stakeholder engagement | Checklist | Yes |
| **Objective of the review** | Objective | Checklist | Yes |
|  | Definition of the question components | Meta-data | Table 1 |
| **Methods** | Protocol | Meta-data | As described in the Materials and Method section |
|  | Deviations from protocol | Checklist | Yes |
| **Searches** | Search strategy | Checklist | Yes |
|  | Search string | Meta-data | Table S2 |
|  | Languages - bibliographic databases | Meta-data | English |
|  | Languages – grey literature | Meta-data | English |
|  | Bibliographic databases | Meta-data | Table S2 |
|  | Web-based search engines | Meta-data | Table S2 |
|  | Organisational websites | Meta-data | Table S2 |
|  | Estimating comprehensiveness of the search | Checklist | Yes |
|  | Search update | Checklist | Yes, Table S2 |
| **Article screening and study inclusion criteria** | Screening strategy | Checklist | Yes |
|  | Inclusion criteria | Checklist | Yes |
| **Meta-data extraction and coding strategy** | Meta-data extraction and coding strategy | Checklist | Yes |
|  | Approaches to missing data | Checklist | Yes |
| **Data synthesis and presentation** | Narrative synthesis strategy | Checklist | Yes |
|  | Knowledge gap and cluster identification strategy | Checklist | Yes |
|  | Demonstrating procedural independence | Checklist | Yes |
| **Results** | Description of review process | Checklist | Yes |
|  | Number of search results | Meta-data | Figure S1 |
|  | Number of search results after duplicate removal | Meta-data | Figure S1 |
|  | Full text screening excludes | Checklist | Yes |
|  | Title screening results | Meta-data | Figure S1 |
|  | Abstract screening results | Meta-data | Figure S1 |
|  | Title and abstract screening results | Meta-data | Figure S1 |
|  | Retrieval results | Meta-data | Figure S1 |
|  | Unobtainable articles | Checklist | No |
|  | Full text screening results | Meta-data | Figure S1 |
|  | Consistency checking: screening | Checklist | Yes |
|  | Narrative synthesis | Checklist | No |
|  | Systematic map database | Checklist | Yes |
| **Discussion** | Discussion | Checklist | Yes |
|  | Limitations of the review | Checklist | Yes |
|  | Limitations of the evidence base | Checklist | Yes |
| **Conclusions** | Knowledge gaps and clusters | Checklist | Yes |
|  | Implications for policy/management | Checklist | Yes |
|  | Implications for research | Checklist | Yes |
| **Declarations** | Competing interests | Checklist | Yes |
