# Supplementary material for: Volcanic-associated ecosystems of the Mediterranean Sea: a systematic map and an interactive tool to support their conservation
Source: PeerJ. 2023 Mar 29;11:e15162. doi: 10.7717/peerj.15162 (PMC10066691; doi:10.7717/peerj.15162)
Supplement: Supplemental Information 2 — The Complex search strings were used on Scopus database and on the platform Web of Science to collect the scientific literature analyzed in the Systematic Map. The Simple search string was used on Google Scholar where the first 100 results were analyzed. [file peerj-11-15162-s002.docx]

**Table S2:**

**Complex and Simple search strings.**

The Complex search strings were used on *Scopus* database and on the platform *Web of Science* to collect the scientific literature analyzed in the Systematic Map. The Simple search string was used on Google Scholar where the first 100 results were analyzed.

| **Search Strings** | **Date** | **Scopus** | **WoS** |
| --- | --- | --- | --- |
| **Complex Search String** |  |  |  |
| TITLE-ABS-KEY(("hydrothermal*" OR "emission*" OR "volcan*" OR "plume*" OR "vent*" OR "seep*" OR "eruption*" OR "acidification" OR "carbon dioxide" OR "pH" OR "CO2" OR "CCS") AND ("sea" OR "ocean" OR "marine") AND ("mediterranean")) AND (LIMIT-TO(LANGUAGE, "English")) | 24/05/2022 | 5,472 |  |
| TS=((hydrothermal* OR emission* OR volcan* OR plume* OR vent* OR seep* OR eruption* OR acidification OR carbon dioxide OR pH OR CO2 OR CCS) AND (sea OR ocean OR marine) AND ("mediterranean")) (limited to English language) | 27/05/2022 |  | 4,838 |
|  |  |  |  |
| **Projects** |  |  |  |
| TITLE-ABS-KEY("mediterranean") AND PUBYEAR AFT 2009 AND FUND-ALL("BIOACID") | 27/05/2022 | 1 |  |
| TS=(mediterranean) AND PY =(2009-2022) AND FT=(BIOACID) | 27/05/2022 |  | 5 |
| TITLE-ABS-KEY("mediterranean") AND PUBYEAR AFT 2007 AND FUND-ALL("EPOCA") | 27/05/2022 | 19 |  |
| TS=(mediterranean) AND PY =(2007-2022) AND FT=(EPOCA) | 27/05/2022 |  | 32 |
| TITLE-ABS-KEY("mediterranean") AND PUBYEAR AFT 2005 AND FUND-ALL("hermes") | 27/05/2022 | 112 |  |
| TS=(mediterranean) AND PY =(2005-2022) AND FT=(HERMES) | 27/05/2022 |  | 170 |
| TITLE-ABS-KEY("mediterranean") AND PUBYEAR AFT 2009 AND FUND-ALL("hermione") | 27/05/2022 | 111 |  |
| TS=(mediterranean) AND PY =(2009-2022) AND FT=(HERMIONE) | 27/05/2022 |  | 201 |
| TITLE-ABS-KEY("mediterranean") AND PUBYEAR AFT 2010 AND FUND-ALL("medsea") | 27/05/2022 | 66 |  |
| TS=(mediterranean) AND PY =(2010-2022) AND FT=(MEDSEA) | 27/05/2022 |  | 109 |
| TITLE-ABS-KEY("mediterranean") AND PUBYEAR AFT 2013 AND FUND-ALL("midas") | 27/05/2022 | 16 |  |
| TS=(mediterranean) AND PY =(2013-2022) AND FT=(MIDAS) | 27/05/2022 |  | 16 |
|  |  |  |  |
| **Simple Search String (search on 27/05/2022) on Google Scholar** |  |  |  |
| "hydrothermal vent" AND "mediterranean" | | | |
