# Supplementary material for: Volcanic-associated ecosystems of the Mediterranean Sea: a systematic map and an interactive tool to support their conservation
Source: PeerJ. 2023 Mar 29;11:e15162. doi: 10.7717/peerj.15162 (PMC10066691; doi:10.7717/peerj.15162)
Supplement: Supplemental Information 3 — The list includes authors, title, year, source title and DOI for the scientific items included in the Systematic Map database (n = 433). [file peerj-11-15162-s003.docx]

**Table S3:**

**Complete list of the scientific items included in the Systematic Map.**

The list includes authors, title, year, source title and doi for the scientific items included in the Systematic Map database (n = 433).

| Authors | Title | Year | Source title | doi |
| --- | --- | --- | --- | --- |
| Aiello G. | New sedimentological and coastal and marine geological data on the Quaternary marine deposits of the Ischia Island (Gulf of Naples, Southern Tyrrhenian Sea, Italy) | 2020 | Geo-Marine Letters | 10.1007/s00367-020-00652-w |
| Aiello G. | New insights on the late Quaternary geologic evolution of the Ischia Island coastal belt based on high-resolution seismic profiles | 2018 | Italian Journal of Geosciences | 10.3301/IJG.2017.19 |
| Aiello G. and Insinga D.D. and Iorio M. and Meo A. and Senatore M.R. | On the occurrence of the Neapolitan Yellow Tuff tephra in the Northern Phlegraean Fields offshore (Eastern Tyrrhenian margin; Italy) | 2017 | Italian Journal of Geosciences | 10.3301/IJG.2017.06 |
| Aiello G. and Marsella E. and Ruggieri S. | Three-dimensional magneto-seismic reconstruction of the 'Torre del Greco' submerged volcanic structure (Naples Bay, Southern Tyrrhenian Sea, Italy): Implications for Vesuvius's marine geophysics and volcanology | 2010 | Near Surface Geophysics | 10.3997/1873-0604.2009037 |
| Aiello, G; Mazzini, I; Parisi, R; Ingrassia, M; Barra, D | Are CO2-rich seafloor pockmarks a suitable environment for ostracod assemblages? The example of the Zannone Giant Pockmark (central-eastern Tyrrhenian) | 2022 | MARINE ECOLOGY-AN EVOLUTIONARY PERSPECTIVE | 10.1111/maec.12698 |
| Akhmanov G.G. and Woodside J.M. | Mud volcanic samples in the context of the Mediterranean Ridge mud diapiric belt | 1998 | Proceedings of the Ocean Drilling Program: Scientific Results | 10.2973/odp.proc.sr.160.045.1998 |
| Albert P.G. and Tomlinson E.L. and Smith V.C. and Di Roberto A. and Todman A. and Rosi M. and Marani M. and Muller W. and Menzies M.A. | Marine-continental tephra correlations: Volcanic glass geochemistry from the Marsili Basin and the Aeolian Islands, Southern Tyrrhenian Sea, Italy | 2012 | Journal of Volcanology and Geothermal Research | 10.1016/j.jvolgeores.2012.03.009 |
| Alfieris D. and Voudouris P. and Spry P.G. | Shallow submarine epithermal Pb-Zn-Cu-Au-Ag-Te mineralization on western Milos Island, Aegean Volcanic Arc, Greece: Mineralogical, geological and geochemical constraints | 2013 | Ore Geology Reviews | 10.1016/j.oregeorev.2013.01.007 |
| Aliani S. and Bortoluzzi G. and Caramanna G. and Raffa F. | Seawater dynamics and environmental settings after November 2002 gas eruption off Bottaro (Panarea, Aeolian Islands, Mediterranean Sea) | 2010 | Continental Shelf Research | 10.1016/j.csr.2010.04.016 |
| Aliani S. and Meloni R. and Dando P.R. | Periodicities in sediment temperature time-series at a marine shallow water hydrothermal vent in Milos Island (Aegean Volcanic arc, Eastern Mediterranean) | 2004 | Journal of Marine Systems | 10.1016/j.jmarsys.2003.11.015 |
| Allen S.R. | Reconstruction of a major caldera-forming eruption from pyroclastic deposit characteristics: Kos Plateau Tuff, eastern Aegean Sea | 2001 | Journal of Volcanology and Geothermal Research | 10.1016/S0377-0273(00)00222-5 |
| Aloisi G. and Pierre C. and Rouchy J.-M. and Foucher J.-P. and Woodside J. | Methane-related authigenic carbonates of Eastern Mediterranean Sea mud volcanoes and their possible relation to gas hydrate destabilisation | 2000 | Earth and Planetary Science Letters | 10.1016/S0012-821X(00)00322-8 |
| Aloisi, G; Wallmann, K; Haese and RR; Saliege and JF | Chemical, biological and hydrological controls on the C-14 content of cold seep carbonate crusts: numerical modeling and implications for convection at cold seeps | 2004 | Chemical Geology | 10.1016/j.chemgeo.2004.07.008 |
| Amend J.P. and Meyer-Dombard D.R. | The shallow-sea hydrothermal system at Vulcano Island (Italy): The 'type locality' for several transformative discoveries in geobiology | 2020 | Italian Journal of Geosciences | 10.3301/IJG.2020.17 |
| Anastasakis G. | The anatomy and provenance of thick volcaniclastic flows in the Cretan Basin, South Aegean Sea | 2007 | Marine Geology | 10.1016/j.margeo.2007.02.019 |
| Andaloro F. and Romeo T. and Renzi M. and Guerranti C. and Perra G. and Consoli P. and Perzia P. and Focardi S.E. | Alteration of potential harmful elements levels in sediments and biota from the central Mediterranean Sea (Aeolian Archipelago) following an episode of intense volcanic activity | 2012 | Environmental Monitoring and Assessment | 10.1007/s10661-011-2242-0 |
| Anzidei M. and Esposito A. and Benini A. | Evidence of active subsidence at Basiluzzo island (Aeolian islands, southern Italy) inferred from a Roman age wharf | 2014 | Quaternary International | 10.1016/j.quaint.2014.03.019 |
| Apostolaki E.T. and Vizzini S. and Hendriks I.E. and Olsen Y.S. | Seagrass ecosystem response to long-term high CO2 in a Mediterranean volcanic vent | 2014 | Marine Environmental Research | 10.1016/j.marenvres.2014.05.008 |
| Appolloni, L; Zeppilli, D; Donnarumma, L; Baldrighi, E; Chianese, E; Russo and GF; Sandulli, R | Seawater Acidification Affects Beta-Diversity of Benthic Communities at a Shallow Hydrothermal Vent in a Mediterranean Marine Protected Area (Underwater Archaeological Park of Baia, Naples, Italy) | 2020 | Diversity | 10.3390/d12120464 |
| Arcadi E., Rastelli E., Tangherlini M., Rizzo C., Mancuso M., Sanfilippo M., Esposito V., Andaloro F., Romeo T. | Shallow-Water Hydrothermal Vents as Natural Accelerators of Bacterial Antibiotic Resistance in Marine Coastal Areas | 2022 | Microorganisms | 10.3390/microorganisms10020479 |
| Azrieli-Tal I. and Matthews A. and Bar-Matthews M. and Almogi-Labin A. and Vance D. and Archer C. and Teutsch N. | Evidence from molybdenum and iron isotopes and molybdenum-uranium covariation for sulphidic bottom waters during Eastern Mediterranean sapropel S1 formation | 2014 | Earth and Planetary Science Letters | 10.1016/j.epsl.2014.02.054 |
| Baggini C. and Issaris Y. and Salomidi M. and Hall-Spencer J. | Herbivore diversity improves benthic community resilience to ocean acidification | 2015 | Journal of Experimental Marine Biology and Ecology | 10.1016/j.jembe.2015.04.019 |
| Baggini C. and Salomidi M. and Voutsinas E. and Bray L. and Krasakopoulou E. and Hall-Spencer J.M. | Seasonality affects macroalgal community response to increases in pCO2 | 2014 | PLoS ONE | 10.1371/journal.pone.0106520 |
| Bagnato E. and Oliveri E. and Acquavita A. and Covelli S. and Petranich E. and Barra M. and Italiano F. and Parello F. and Sprovieri M. | Hydrochemical mercury distribution and air-sea exchange over the submarine hydrothermal vents off-shore Panarea Island (Aeolian arc, Tyrrhenian Sea) | 2017 | Marine Chemistry | 10.1016/j.marchem.2017.04.003 |
| Baldrighi, E; Zeppille, D; Appolloni, L; Donnarumma, L; Chianese, E; Russo and GF; Sandulli, R | Meiofaunal communities and nematode diversity characterizing the Secca delle Fumose shallow vent area (Gulf of Naples, Italy) | 2020 | PeerJ | 10.7717/peerj.9058 |
| Banister R.B. and Schwarz M.T. and Fine M. and Ritchie K.B. and Muller E.M. | Instability and Stasis Among the Microbiome of Seagrass Leaves, Roots and Rhizomes, and Nearby Sediments Within a Natural pH Gradient | 2021 | Microbial Ecology | 10.1007/s00248-021-01867-9 |
| Barca D. and Trua T. | Magma emplacement at anomalous spreading ridge: Constraints due to plagioclase crystals from basalts of Marsili seamount (Southern Tyrrhenian back-arc) | 2012 | Journal of Volcanology and Geothermal Research | 10.1016/j.jvolgeores.2012.06.021 |
| Barruffo A. and Ciaralli L. and Ardizzone G. and Gambi M.C. and Casoli E. | Ocean acidification and mollusc settlement in posidonia oceanica meadows: Does the seagrass buffer lower ph effects at co2 vents? | 2021 | Diversity | 10.3390/d13070311 |
| Basso L. and Hendriks I.E. and RodrÃ­guez-Navarro A.B. and Gambi M.C. and Duarte C.M. | Extreme pH Conditions at a Natural CO2 Vent System (Italy) Affect Growth, and Survival of Juvenile Pen Shells (Pinna nobilis) | 2015 | Estuaries and Coasts | 10.1007/s12237-014-9936-9 |
| Bayon G. and Loncke L. and DuprÃ© S. and Caprais J.-C. and Ducassou E. and Duperron S. and Etoubleau J. and Foucher J.-P. and Fouquet Y. and Gontharet S. and Henderson G.M. and Huguen C. and Klaucke I. and Mascle J. and Migeon S. and Olu-Le Roy K. and OndrÃ©as H. and Pierre C. and Sibuet M. and Stadnitskaia A. and Woodside J. | Multi-disciplinary investigation of fluid seepage on an unstable margin: The case of the Central Nile deep sea fan | 2009 | Marine Geology | 10.1016/j.margeo.2008.10.008 |
| Bayon, G; Dupre, S; Ponzevera, E; Etoubleau, J; Cheron, S; Pierre, C; Mascle, J; Boetius, A; de Lange and GJ | Formation of carbonate chimneys in the Mediterranean Sea linked to deep-water oxygen depletion | 2013 | Nature Geoscience | 10.1038/NGEO1888 |
| Bayona J.M. and Monjonell A. and Miquel J.C. and Fowler S.W. and Albaigés J. | Biogeochemical characterization of participate organic matter from a coastal hydrothermal vent zone in the Aegean Sea | 2002 | Organic Geochemistry | 10.1016/S0146-6380(02)00175-4 |
| Beccaluva L. and Gabbianelli G. and Lucchini F. and Rossi P.L. and Savelli C. | Petrology and K Ar ages of volcanics dredged from the Eolian seamounts: implications for geodynamic evolution of the southern Tyrrhenian basin | 1985 | Earth and Planetary Science Letters | 10.1016/0012-821X(85)90021-4 |
| Bellec, L; Cambon-Bonavita and MA; Durand, L; Aube, J; Gayet, N; Sandulli, R; Brandily, C; Zeppilli, D | Microbial Communities of the Shallow-Water Hydrothermal Vent Near Naples, Italy, and Chemosynthetic Symbionts Associated With a Free-Living Marine Nematode | 2020 | Frontiers in Microbiology | 10.3389/fmicb.2020.02023 |
| Bellissimo G. and Rull Lluch J. and Tomasello A. and Calvo S. | The community of Cystoseira brachycarpa J. Agardh emend. Giaccone (Fucales, Phaeophyceae) in a shallow hydrothermal vent area of the Aeolian Islands (Tyrrhenian Sea, Italy) | 2014 | Plant Biosystems | 10.1080/11263504.2013.778350 |
| Biagi E. and Caroselli E. and Barone M. and Pezzimenti M. and Teixido N. and Soverini M. and Rampelli S. and Turroni S. and Gambi M.C. and Brigidi P. and Goffredo S. and Candela M. | Patterns in microbiome composition differ with ocean acidification in anatomic compartments of the Mediterranean coral Astroides calycularis living at CO2 vents | 2020 | Science of the Total Environment | 10.1016/j.scitotenv.2020.138048 |
| Bianchi C.N. and Dando P.R. and Morri C. | Increased diversity of sessile epibenthos at subtidal hydrothermal vents: Seven hypotheses based on observations at Milos Island, Aegean Sea | 2011 | Advances in Oceanography and Limnology | 10.1080/19475721.2011.565804 |
| Bianchi C.N. and Morri C. | Serpuloidea (Annelida: Polychaeta) from Milos, an island in the Aegean Sea with submarine hydrothermalism | 2000 | Journal of the Marine Biological Association of the United Kingdom | 10.1017/S0025315400001831 |
| Blinova V.N. and Comas M.C. and Ivanov M.K. and Poludetkina E.N. and Matveeva T.V. | Active mud volcanism in the West Alboran Basin: Geochemical evidence of hydrocarbon seepage | 2011 | Marine and Petroleum Geology | 10.1016/j.marpetgeo.2011.06.001 |
| Boatta F., D'Alessandro W. and Gagliano A.L. and Liotta M. and Milazzo M. and Rodolfo-Metalpa R. and Hall-Spencer J.M. and Parello F. | Geochemical survey of Levante Bay, Vulcano Island (Italy), a natural laboratory for the study of ocean acidification | 2013 | Marine Pollution Bulletin | 10.1016/j.marpolbul.2013.01.029 |
| Boggemann M. and Bienhold C. and Gaudron S.M. | A new species of Glyceridae (Annelida: "Polychaeta") recovered from organic substrate experiments at cold seeps in the eastern Mediterranean Sea | 2012 | Marine Biodiversity | 10.1007/s12526-011-0091-2 |
| Boisson F. and Miquel J.-C. and Cotret O. and Fowler S.W. | 210Po and 210Pb cycling in a hydrothermal vent zone in the coastal Aegean Sea | 2001 | Science of the Total Environment | 10.1016/S0048-9697(01)00840-3 |
| Borda, E; Kudenov and JD; Bienhold, C; Rouse and GW | Towards a revised Amphinomidae (Annelida, Amphinomida): description and affinities of a new genus and species from the Nile Deep-sea Fan, Mediterranean Sea | 2012 | Zoologica Scripta | 10.1111/j.1463-6409.2012.00529.x |
| Bortoluzzi G. and Romeo T. and La Cono V. and La Spada G. and Smedile F. and Esposito V. and Sabatino G. and Di Bella M. and Canese S. and Scotti G. and Bo M. and Giuliano L. and Jones D. and Golyshin P.N. and Yakimov M.M. and Andaloro F. | Ferrous iron- and ammonium-rich diffuse vents support habitat-specific communities in a shallow hydrothermal field off the Basiluzzo Islet (Aeolian Volcanic Archipelago). | 2017 | Geobiology | 10.1111/gbi.12237 |
| BostrÃ¶m K. and Widenfalk L. | The origin of iron-rich muds at the Kameni Islands, Santorini, Greece | 1984 | Chemical Geology | 10.1016/0009-2541(84)90015-9 |
| Botz R. and StÃ¼ben D. and Winckler G. and Bayer R. and Schmitt M. and Faber E. | Hydrothermal gases offshore Milos island, Greece | 1996 | Chemical Geology | 10.1016/0009-2541(96)00023-X |
| Bouloubassi I. and Aloisi G. and Pancost R.D. and Hopmans E. and Pierre C. and Sinninghe Damsté J.S. | Archaeal and bacterial lipids in authigenic carbonate crusts from eastern Mediterranean mud volcanoes | 2006 | Organic Geochemistry | 10.1016/j.orggeochem.2005.11.005 |
| Bouraoui H. and Rebib H. and Aissa M.B. and Touzel J.P., O'donohue M. and Manai M. | Paenibacillus marinum sp. nov., a thermophilic xylanolytic bacterium isolated from a marine hot spring in Tunisia | 2013 | Journal of Basic Microbiology | 10.1002/jobm.201200275 |
| Bravakos P. and Mandalakis M. and Nomikou P. and Anastasiou T.I. and Kristoffersen J.B. and Stavroulaki M. and Kilias S. and Kotoulas G. and Magoulas A. and Polymenakou P.N. | Genomic adaptation of Pseudomonas strains to acidity and antibiotics in hydrothermal vents at Kolumbo submarine volcano, Greece | 2021 | Scientific Reports | 10.1038/s41598-020-79359-y |
| Bray, L; Pancucci-Papadopulou and MA; Hall-Spencer and JM | Sea urchin response to rising pCO(2) shows ocean acidification may fundamentally alter the chemistry of marine skeletons | 2014 | Mediterranean Marine Science | 10.12681/mms.579 |
| Brewer P.G. | A different ocean acidification hazard-the kolumbo submarine volcano example | 2013 | Geology | 10.1130/focus092013.1 |
| Brinkhoff T. and Sievert S.M. and Kuever J. and Muyzer G. | Distribution and diversity of sulfur-oxidizing Thiomicrospira spp. at a shallow-water hydrothermal vent in the Aegean Sea (Milos, Greece) | 1999 | Applied and Environmental Microbiology | 10.1128/aem.65.9.3843-3849.1999 |
| Brissac T. and Rodrigues C.F. and Gros O. and Duperron S. | Characterization of bacterial symbioses in Myrtea sp. (Bivalvia: Lucinidae) and Thyasira sp. (Bivalvia: Thyasiridae) from a cold seep in the Eastern Mediterranean | 2011 | Marine Ecology | 10.1111/j.1439-0485.2010.00413.x |
| Bruno P.P.G. and de Alteriis G. and Florio G. | The western undersea section of the Ischia volcanic complex (Italy, Tyrrhenian sea) inferred by marine geophysical data | 2002 | Geophysical Research Letters | 10.1029/2001gl013904 |
| Cagatay M.N., Yildiz G. and Bayon G. and Ruffine L. and Henry P. | Seafloor authigenic carbonate crusts along the submerged part of the North Anatolian Fault in the Sea of Marmara: Mineralogy, geochemistry, textures and genesis | 2018 | Deep-Sea Research Part II: Topical Studies in Oceanography | 10.1016/j.dsr2.2017.09.003 |
| Calosi P. and Rastrick S.P.S. and Lombardi C. and de Guzman H.J. and Davidson L. and Jahnke M. and Giangrande A. and Hardege J.D. and Schulze A. and Spicer J.I. and Gambi M.-C. | Adaptation and acclimatization to ocean acidification in marine ectotherms: An in situ transplant experiment with polychaetes at a shallow CO2 vent system | 2013 | Philosophical Transactions of the Royal Society B: Biological Sciences | 10.1098/rstb.2012.0444 |
| Camerlenghi A. and Cita M.B. and Vedova B.D. and Fusi N. and Mirabile L. and Pellis G. | Geophysical evidence of mud diapirism on the Mediterranean Ridge accretionary complex | 1995 | Marine Geophysical Researches | 10.1007/BF01203423 |
| Camilli R. and Nomikou P. and EscartÃ­n J. and Ridao P. and Mallios A. and Kilias S.P. and Argyraki A. and Andreani M. and Ballu V. and Campos R. and Deplus C. and Gabsi T. and Garcia R. and Gracias N. and HurtÃ³s N. and MagÃ­ L., MÃ©vel C. and Moreira M. and Palomeras N. and Pot O. and Ribas D. and RuziÃ© L. and Sakellariou D. | The Kallisti Limnes, carbon dioxide-accumulating subsea pools | 2015 | Scientific Reports | 10.1038/srep12152 |
| Cangemi M. and Di Leonardo R. and Bellanca A. and Cundy A. and Neri R. and Angelone M. | Geochemistry and mineralogy of sediments and authigenic carbonates from the Malta Plateau, Strait of Sicily (Central Mediterranean): Relationships with mud/fluid release from a mud volcano system | 2010 | Chemical Geology | 10.1016/j.chemgeo.2010.06.014 |
| Cantner K. and Carey S. and Nomikou P. | Integrated volcanologic and petrologic analysis of the 1650AD eruption of Kolumbo submarine volcano, Greece | 2014 | Journal of Volcanology and Geothermal Research | 10.1016/j.jvolgeores.2013.10.004 |
| Caprais and JC; Lanteri, N; Crassous, P; Noel, P; Bignon, L; Rousseaux, P; Pignet, P; Khripounoff, A | A new CALMAR benthic chamber operating by submersible: First application in the cold-seep environment of Napoli mud volcano (Mediterranean Sea) | 2010 | Limnology and Oceanography-Methods | 10.4319/lom.2010.8.304 |
| Caracausi A. and Ditta M. and Italiano F. and Longo M. and Nuccio P.M. and Paonita A. | Massive submarine gas output during the volcanic unrest off Panarea Island (Aeolian arc, Italy): Inferences for explosive conditions | 2005 | Geochemical Journal | 10.2343/geochemj.39.459 |
| Caramanna G. and Espa S. and Bouché V. | Study of the environmental effects of submarine CO2-rich emissions by means of scientific diving techniques (Panarea Island - Italy) | 2010 | Underwater Technology | 10.3723/ut.29.079 |
| Caramanna G. and Fietzek P. and Maroto-Valer M. | Monitoring techniques of a natural analogue for sub-seabed CO2 leakages | 2011 | Energy Procedia | 10.1016/j.egypro.2011.02.245 |
| Caramanna G. and Voltattorni N. and Maroto-Valer M.M. | Eight years of research on a marine natural analogue for sub-seabed CO 2 storage seepage | 2011 | OCEANS 2011 IEEE - Spain | 10.1109/Oceans-Spain.2011.6003452 |
| Caramanna G. and Voltattorni N. and Mercedes Maroto-Valer M. | Is Panarea Island (Italy) a valid and cost-effective natural laboratory for the development of detection and monitoring techniques for submarine CO 2 seepage? | 2011 | Greenhouse Gases: Science and Technology | 10.1002/ghg.28 |
| Caratori Tontini F. and Bortoluzzi G. and Carmisciano C. and Cocchi L. and De Ronde C.E.J. and Ligi M. and Muccini F. | Near-bottom magnetic signatures of submarine hydrothermal systems at Marsili and Palinuro volcanoes, southern Tyrrhenian Sea, Italy | 2014 | Economic Geology | 10.2113/econgeo.109.8.2119 |
| Caratori Tontini F. and Cocchi L. and Muccini F. and Carmisciano C. and Marani M. and Bonatti E. and Ligi M. and Boschi E. | Potential-field modeling of collapse-prone submarine volcanoes in the southern Tyrrhenian Sea (Italy) | 2010 | Geophysical Research Letters | 10.1029/2009GL041757 |
| Carbonne C. and TeixidÃ³ N. and Moore B. and Mirasole A. and Guttierez T. and Gattuso J.-P. and Comeau S. | Two temperate corals are tolerant to low pH regardless of previous exposure to natural CO2 vents | 2021 | Limnology and Oceanography | 10.1002/lno.11942 |
| Carey S. and Nomikou P. and Bell K.C. and Lilley M. and Lupton J. and Roman C. and Stathopoulou E. and Bejelou K. and Ballard R. | Co2 degassing from hydrothermal vents at kolumbo submarine volcano, greece, and the accumulation of acidic crater water | 2013 | Geology | 10.1130/G34286.1 |
| Carlier A. and Ritt B. and Rodrigues C.F. and Sarrazin J. and Olu K. and Grall J. and Clavier J. | Heterogeneous energetic pathways and carbon sources on deep eastern Mediterranean cold seep communities | 2010 | Marine Biology | 10.1007/s00227-010-1518-1 |
| Caroselli E. and Gizzi F. and Prada F. and Marchini C. and Airi V. and Kaandorp J. and Falini G. and Dubinsky Z. and Goffredo S. | Low and variable pH decreases recruitment efficiency in populations of a temperate coral naturally present at a CO2 vent | 2019 | Limnology and Oceanography | 10.1002/lno.11097 |
| Casalbore D. and Ingrassia M. and Pierdomenico M. and Beaubien S.E. and Martorelli E. and Bigi S. and Ivaldi R. and DeMarte M. and Chiocci F.L. | Morpho-acoustic characterization of a shallow-water mud volcano offshore Scoglio d'Affrica (Northern Tyrrhenian Sea) responsible for a violent gas outburst in 2017 | 2020 | Marine Geology | 10.1016/j.margeo.2020.106277 |
| Casalbore D. and Romagnoli C. and Chiocci F. and Frezza V. | Morpho-sedimentary characteristics of the volcaniclastic apron around Stromboli volcano (Italy) | 2010 | Marine Geology | 10.1016/j.margeo.2010.01.004 |
| Casas D. and Ercilla G. and Lykousis V. and Ioakim C. and Perissoratis C. | Physical properties and their relationship to sedimentary processes and texture in sediments from mud volcanoes in the Anaximander Mountains (Eastern Mediterranean) | 2006 | Scientia Marina | 10.3989/scimar.2006.70n4643 |
| Cattano C. and Calò A. and Di Franco A. and Firmamento R. and Quattrocchi F. and Sdiri K. and Guidetti P. and Milazzo M. | Ocean acidification does not impair predator recognition but increases juvenile growth in a temperate wrasse off CO2 seeps | 2017 | Marine Environmental Research | 10.1016/j.marenvres.2017.10.013 |
| Celis-PlÃ¡ P.S.M. and Hall-Spencer J.M. and Horta P.A. and Milazzo M. and Korbee N. and Cornwall C.E. and Figueroa F.L. | Macroalgal responses to ocean acidification depend on nutrient and light levels | 2015 | Frontiers in Marine Science | 10.3389/fmars.2015.00026 |
| Ceramicola S. and Praeg D. and Cova A. and Accettella D. and Zecchin M. | Seafloor distribution and last glacial to postglacial activity of mud volcanoes on the Calabrian accretionary prism, Ionian Sea | 2014 | Geo-Marine Letters | 10.1007/s00367-013-0354-y |
| Charlou J.L. and Donval J.P. and Zitter T. and Roy N. and Jean-Baptiste P. and Foucher J.P. and Woodside J. | Evidence of methane venting and geochemistry of brines on mud volcanoes of the eastern Mediterranean Sea | 2003 | Deep-Sea Research Part I: Oceanographic Research Papers | 10.1016/S0967-0637(03)00093-1 |
| Chauhan, A; Pathak, A; Rodolfo-Metalpa, R; Milazzo, M; Green and SJ; Hall-Spencer and JM | Metagenomics Reveals Planktonic Bacterial Community Shifts across a Natural CO2 Gradient in the Mediterranean Sea | 2015 | MICROBIOLOGY RESOURCE ANNOUNCEMENTS | 10.1128/genomeA.01543-14 |
| Chevalier N. and Bouloubassi I. and Birgel D. and Taphanel M.-H., López-García P. | Microbial methane turnover at Marmara Sea cold seeps: A combined 16S rRNA and lipid biomarker investigation | 2013 | Geobiology | 10.1111/gbi.12014 |
| Chevalier, N; Bouloubassi, I; Birgel, D; Cremiere, A; Taphanel and MH; Pierre, C | Authigenic carbonates at cold seeps in the Marmara Sea (Turkey): A lipid biomarker and stable carbon and oxygen isotope investigation | 2011 | Marine Geology | 10.1016/j.margeo.2011.08.005 |
| Chiocci F.L. and Orlando L. | Lowstand terraces on Tyrrhenian Sea steep continental slopes | 1996 | Marine Geology | 10.1016/0025-3227(96)00023-0 |
| Christopoulou M.E. and Mertzimekis T.J. and Nomikou P. and Papanikolaou D. and Carey S. and Mandalakis M. | Influence of hydrothermal venting on water column properties in the crater of the Kolumbo submarine volcano, Santorini volcanic field (Greece) | 2016 | Geo-Marine Letters | 10.1007/s00367-015-0429-z |
| Cigliano M. and Gambi M.C. and Rodolfo-Metalpa R. and Patti F.P. and Hall-Spencer J.M. | Effects of ocean acidification on invertebrate settlement at volcanic CO2 vents | 2010 | Marine Biology | 10.1007/s00227-010-1513-6 |
| Cita M.B. and Erba E. and Lucchi R. and Pott M. and Van Der Meer R. and Nieto L. | Stratigraphy and sedimentation in the Mediterranean Ridge diapiric belt | 1996 | Marine Geology | 10.1016/0025-3227(96)00157-0 |
| Cita M.B. and Woodside J.M. and Ivanov M.K. and Kidd R.B. and Limonov A.F. and Scientific Staff of Cruise TTR3 - Leg 2 | Fluid venting from a mud volcano in the Mediterranean Ridge Diapiric Belt | 1995 | Terra Nova | 10.1111/j.1365-3121.1995.tb00541.x |
| Civile D. and Lodolo E. and Caffau M. and Baradello L. and Ben-Avraham Z. | Anatomy of a submerged archipelago in the Sicilian Channel (central Mediterranean Sea) | 2016 | Geological Magazine | 10.1017/S0016756815000485 |
| Cocchi L. and Caratori Tontini F. and Muccini F. and Marani M.P. and Bortoluzzi G. and Carmisciano C. | Chronology of the transition from a spreading ridge to an accretional seamount in the Marsili backarc basin (Tyrrhenian Sea) | 2009 | Terra Nova | 10.1111/j.1365-3121.2009.00891.x |
| Cocito S. and Bianchi C.N. and Morri C. and Peirano A. | First survey of sessile communities on subtidal rocks in an area with hydrothermal vents: Milos Island, Aegean Sea | 2000 | Hydrobiologia | 10.1023/A:1003991117108 |
| Coleman D.F. and Ballard R.D. | A highly concentrated region of cold hydrocarbon seeps in the Southeastern Mediterranean Sea | 2001 | Geo-Marine Letters | 10.1007/s003670100079 |
| Coltelli M. and Cavallaro D., D’Anna G., D’Alessandro A. and Grassa F. and Mangano G. and Patanè D. and Gresta S. | Exploring the submarine graham bank in the sicily channel | 2016 | Annals of Geophysics | 10.4401/ag-6929 |
| Consoli P. and Esposito V. and Battaglia P. and Perzia P. and Scotti G., D'Alessandro M. and Canese S. and Andaloro F. and Romeo T. | Marine litter pollution associated with hydrothermal sites in the Aeolian archipelago (western Mediterranean Sea) | 2021 | Science of the Total Environment | 10.1016/j.scitotenv.2021.144968 |
| Conte A.M. and Caramanna G. | Preliminary characterisation of a shallow water hydrothermal sulphide deposit recovered by scientific divers (Aeolian islands, southern Tyrrhenian Sea) | 2010 | Underwater Technology | 10.3723/ut.29.109 |
| Conte A.M. and Di Bella L. and Ingrassia M. and Perinelli C. and Martorelli E. | Alteration and mineralization products of the zannone giant pockmark (Zannone hydrothermal field, central tyrrhenian sea) | 2020 | Minerals | 10.3390/min10070581 |
| Conte A.M. and Martorelli E. and Calarco M. and Sposato A. and Perinelli C. and Coltelli M. and Chiocci F.L. | The 1891 submarine eruption offshore Pantelleria Island (Sicily Channel, Italy): Identification of the vent and characterization of products and eruptive style | 2014 | Geochemistry, Geophysics, Geosystems | 10.1002/2014GC005238 |
| Conte A.M. and Perinelli C. and Bianchini G. and Natali C. and Martorelli E. and Chiocci F.L. | New insights on the petrology of submarine volcanics from the Western Pontine Archipelago (Tyrrhenian Sea, Italy) | 2016 | Journal of Volcanology and Geothermal Research | 10.1016/j.jvolgeores.2016.08.005 |
| Conte A.M. and Perinelli C. and Bosman A. and Castorina F. and Conti A. and Cuffaro M. and Di Vincenzo G. and Martorelli E. and Bigi S. | Tectonics, Dynamics, and Plio-Pleistocene Magmatism in the Central Tyrrhenian Sea: Insights From the Submarine Transitional Basalts of the Ventotene Volcanic Ridge (Pontine Islands, Italy) | 2020 | Geochemistry, Geophysics, Geosystems | 10.1029/2020GC009346 |
| Corinaldesi C. and Dell'Anno A. and Danovaro R. | Viral infections stimulate the metabolism and shape prokaryotic assemblages in submarine mud volcanoes | 2012 | ISME Journal | 10.1038/ismej.2011.185 |
| Cornwall C.E. and Revill A.T. and Hall-Spencer J.M. and Milazzo M. and Raven J.A. and Hurd C.L. | Inorganic carbon physiology underpins macroalgal responses to elevated CO2 | 2017 | Scientific Reports | 10.1038/srep46297 |
| Corselli C. and Basso D. | First evidence of benthic communities based on chemosynthesis on the Napoli mud volcano (eastern Mediterranean) | 1996 | Marine Geology | 10.1016/0025-3227(95)00163-8 |
| CrÃ©miÃ¨re A. and Bayon G. and Ponzevera E. and Pierre C. | Paleo-environmental controls on cold seep carbonate authigenesis in the Sea of Marmara | 2013 | Earth and Planetary Science Letters | 10.1016/j.epsl.2013.06.029 |
| Cronan D.S. | Hydrothermal Mineralizing Processes and Associated Sedimentation in the Santorini Hydrothermal Embayments | 2000 | Marine Georesources and Geotechnology | 10.1080/10641190009353783 |
| Cronan D.S. and Varnavas S. and Perissoratis C. | Hydrothermal sedimentation in the caldera of Santorini, Hellenic Volcanic Arc | 1995 | Terra Nova | 10.1111/j.1365-3121.1995.tb00696.x |
| Cronin B.T. and Ivanov M.K. and Limonov A.F. and Egorov A. and Akhmanov G.G. and Akhmetjanov A.M. and Kozlova E. | New discoveries of mud volcanoes on the eastern Mediterranean Ridge | 1997 | Journal of the Geological Society | 10.1144/gsjgs.154.2.0173 |
| Cuffaro M. and Billi A. and Bigi S. and Bosman A. and Caruso C.G. and Conti A. and Corbo A. and Costanza A., D'Anna G. and Doglioni C. and Esestime P. and Fertitta G. and Gasperini L. and Italiano F. and Lazzaro G. and Ligi M. and Longo M. and Martorelli E. and Petracchini L. and Petricca P. and Polonia A. and Sgroi T. | The Bortoluzzi Mud Volcano (Ionian Sea, Italy) and its potential for tracking the seismic cycle of active faults | 2019 | Solid Earth | 10.5194/se-10-741-2019 |
| Cuffaro M. and Martorelli E. and Bosman A. and Conti A. and Bigi S. and Muccini F. and Cocchi L. and Ligi M. and Bortoluzzi G. and Scrocca D. and Canese S. and Chiocci F.L. and Conte A.M. and Doglioni C. and Perinelli C. | The Ventotene Volcanic Ridge: a newly explored complex in the central Tyrrhenian Sea (Italy) | 2016 | Bulletin of Volcanology | 10.1007/s00445-016-1081-9 |
| DÃ¤hlmann A. and de Lange G.J. | Fluid-sediment interactions at Eastern Mediterranean mud volcanoes: A stable isotope study from ODP Leg 160 | 2003 | Earth and Planetary Science Letters | 10.1016/S0012-821X(03)00227-9 |
| Dando P.R. and Aliani S. and Arab H. and Bianchi C.N. and Brehmer M. and Cocito S. and Fowler S.W. and Gundersen J. and Hooper L.E., Kölbl R. and Kuever J. and Linke P. and Makropoulos K.C. and Meloni R. and Miquel J.-C. and Morri C., Müller S. and Robinson C. and Schlesner H. and Sievert S. and Stöhr R. and Stüben D. and Thomm M. and Varnavas S.P. and Ziebis W. | Hydrothermal studies in the Aegean sea | 2000 | Physics and Chemistry of the Earth, Part B: Hydrology, Oceans and Atmosphere | 10.1016/S1464-1909(99)00112-4 |
| Dando P.R. and Hughes J.A. and Leahy Y. and Niven S.J. and Taylor L.J. and Smith C. | Gas venting rates from submarine hydrothermal areas around the island of Milos, Hellenic Volcanic Arc | 1995 | Continental Shelf Research | 10.1016/0278-4343(95)80002-U |
| De Alteriis G. and Insinga D. and Morabito S. and Morra V. and Chiocci F.L. and Terrasi F. and Lubritto C. and Di Benedetto C. and Pazzanese M. | Age of submarine debris avalanches and tephrostratigraphy offshore Ischia Island, Tyrrhenian Sea, Italy | 2010 | Marine Geology | 10.1016/j.margeo.2010.08.004 |
| De Biasi A.M. and Aliani S. | Shallow-water hydrothermal vents in the Mediterranean sea: Stepping stones for Lessepsian migration? | 2003 | Hydrobiologia | 10.1023/B:HYDR.0000008484.91786.e8 |
| De Biasi A.M. and Bianchi C.N. and Aliani S. and Cocito S. and Peirano A. and Dando P.R. and Morri C. | Epibenthic communities in a marine shallow area with hydrothermal vents (Milos Island, Aegean Sea) | 2004 | Chemistry and Ecology | 10.1080/02757540310001629152 |
| De Capitani L. and Cita M.B. | The 'marker-bed' of the Mediterranean Ridge diapiric belt: Geochemical characteristics | 1996 | Marine Geology | 10.1016/0025-3227(95)00162-X |
| De Groote A. and Hauquier F. and Vanreusel A. and Derycke S. | Population genetic structure in Sabatieria (Nematoda) reveals intermediary gene flow and admixture between distant cold seeps from the Mediterranean Sea | 2017 | BMC Evolutionary Biology | 10.1186/s12862-017-1003-2 |
| Dekov V.M. and Kamenov G.D. and Abrasheva M.D. and Capaccioni B. and Munnik F. | Mineralogical and geochemical investigation of seafloor massive sulfides from Panarea Platform (Aeolian Arc, Tyrrhenian Sea) | 2013 | Chemical Geology | 10.1016/j.chemgeo.2012.10.048 |
| Dekov V.M. and Kamenov G.D. and Savelli C. and Stummeyer J. | Anthropogenic Pb component in hydrothermal ochres from Marsili Seamount (Tyrrhenian Sea) | 2006 | Marine Geology | 10.1016/j.margeo.2006.03.003 |
| Dekov V.M. and Kamenov G.D. and Savelli C. and Stummeyer J. and Marchig V. | Origin of basal dolomitic claystone in the Marsili Basin, Tyrrhenian Sea | 2007 | Marine Geology | 10.1016/j.margeo.2006.10.021 |
| Dekov V.M. and Kamenov G.D. and Savelli C. and Stummeyer J. and Thiry M. and Shanks W.C. and Willingham A.L. and Boycheva T.B. and Rochette P. and Kuzmann E. and Fortin D., Vértes A. | Metalliferous sediments from Eolo Seamount (Tyrrhenian Sea): Hydrothermal deposition and re-deposition in a zone of oxygen depletion | 2009 | Chemical Geology | 10.1016/j.chemgeo.2009.03.023 |
| Dekov V.M. and Kamenov G.D. and Stummeyer J. and Thiry M. and Savelli C. and Shanks W.C. and Fortin D. and Kuzmann E., Vértes A. | Hydrothermal nontronite formation at Eolo Seamount (Aeolian volcanic arc, Tyrrhenian Sea) | 2007 | Chemical Geology | 10.1016/j.chemgeo.2007.08.006 |
| Del Pasqua M. and Gambi M.C. and Caricato R. and Lionetto M.G. and Giangrande A. | Effects of short-term and long-term exposure to ocean acidification on carbonic anhydrase activity and morphometric characteristics in the invasive polychaete Branchiomma boholense (Annelida: Sabellidae): A case-study from a CO2 vent system | 2019 | Marine Environmental Research | 10.1016/j.marenvres.2019.01.011 |
| Deyhle A. and Kopf A.J. and Aloisi G. | Boron and boron isotopes as tracers for diagenetic reactions and depth of mobilization, using muds and authigenic carbonates from eastern Mediterranean mud volcanoes | 2003 | Geological Society Special Publication | 10.1144/GSL.SP.2003.216.01.32 |
| Di Bella L. and Ingrassia M. and Frezza V. and Chiocci F.L. and Martorelli E. | The response of benthic meiofauna to hydrothermal emissions in the Pontine Archipelago, Tyrrhenian Sea (central Mediterranean Basin) | 2016 | Journal of Marine Systems | 10.1016/j.jmarsys.2016.08.002 |
| Di Bella L. and Ingrassia M. and Frezza V. and Chiocci F.L. and Pecci R. and Bedini R. and Martorelli E. | Spiculosiphon oceana (Foraminifera) a new bio-indicator of acidic environments related to fluid emissions of the Zannone Hydrothermal Field (central Tyrrhenian Sea) | 2018 | Marine Environmental Research | 10.1016/j.marenvres.2018.02.015 |
| Di Giglio S. and Spatafora D. and Milazzo M., M'Zoudi S. and Zito F. and Dubois P. and Costa C. | Are control of extracellular acid-base balance and regulation of skeleton genes linked to resistance to ocean acidification in adult sea urchins? | 2020 | Science of the Total Environment | 10.1016/j.scitotenv.2020.137443 |
| Di Roberto A. and Rosi M. and Bertagnini A. and Marani M.P. and Gamberi F. and Del Principe A. | Deep water gravity core from the Marsili Basin (Tyrrhenian Sea) records Pleistocenic-Holocenic explosive events and instability of the Aeolian Archipelago, (Italy) | 2008 | Journal of Volcanology and Geothermal Research | 10.1016/j.jvolgeores.2008.01.009 |
| Dias B.B. and Hart M.B. and Smart C.W. and Hall-Spencer J.M. | Modern seawater acidification: The response of foraminifera to high-CO2 conditions in the Mediterranean Sea | 2010 | Journal of the Geological Society | 10.1144/0016-76492010-050 |
| Dimitrov L. and Woodside J. | Deep sea pockmark environments in the eastern Mediterranean | 2003 | Marine Geology | 10.1016/S0025-3227(02)00692-8 |
| Donnarumma L. and Appolloni L. and Chianese E. and Bruno R. and Baldrighi E. and Guglielmo R. and Russo G.F. and Zeppilli D. and Sandulli R. | Environmental and Benthic Community Patterns of the Shallow Hydrothermal Area of Secca Delle Fumose (Baia, Naples, Italy) | 2019 | Frontiers in Marine Science | 10.3389/fmars.2019.00685 |
| Donnarumma L. and Lombardi C. and Cocito S. and Gambi M.C. | Settlement pattern of Posidonia oceanica epibionts along a gradient of ocean acidification: An approach with mimics | 2014 | Mediterranean Marine Science | 10.12681/mms.677 |
| Drab L. and Carlut J. and Hubert-Ferrari A. and Martinez P. and LePoint G. and El Ouahabi M. | Paleomagnetic and geochemical record from cores from the Sea of Marmara, Turkey: Age constraints and implications of sapropelic deposition on early diagenesis | 2015 | Marine Geology | 10.1016/j.margeo.2014.12.002 |
| Duperron S. and De Beer D. and Zbinden M. and Boetius A. and Schipani V. and Kahil N. and Gaill F. | Molecular characterization of bacteria associated with the trophosome and the tube of Lamellibrachia sp., a siboglinid annelid from cold seeps in the eastern Mediterranean | 2009 | FEMS Microbiology Ecology | 10.1111/j.1574-6941.2009.00724.x |
| Duperron S. and Fiala-MÃ©dioni A. and Caprais J.-C. and Olu K. and Sibuet M. | Evidence for chemoautotrophic symbiosis in a Mediterranean cold seep clam (Bivalvia: Lucinidae): comparative sequence analysis of bacterial 16S rRNA, APS reductase and RubisCO genes | 2007 | FEMS Microbiology Ecology | 10.1111/j.1574-6941.2006.00194.x |
| DuprÃ© S. and Mascle J. and Foucher J.-P. and Harmegnies F. and Woodside J. and Pierre C. | Warm brine lakes in craters of active mud volcanoes, Menes caldera off NW Egypt: Evidence for deep-rooted thermogenic processes | 2014 | Geo-Marine Letters | 10.1007/s00367-014-0367-1 |
| DuprÃ© S. and Woodside J. and Foucher J.-P. and de Lange G. and Mascle J. and Boetius A. and Mastalerz V. and Stadnitskaia A. and OndrÃ©as H. and Huguen C. and HarmÃ©gnies F. and Gontharet S. and Loncke L. and Deville E. and Niemann H. and Omoregie E. and Olu-Le Roy K. and Fiala-Medioni A., DÃ¤hlmann A. and Caprais J.-C. and Prinzhofer A. and Sibuet M. and Pierre C. and DamstÃ© J.S. | Seafloor geological studies above active gas chimneys off Egypt (Central Nile Deep Sea Fan) | 2007 | Deep-Sea Research Part I: Oceanographic Research Papers | 10.1016/j.dsr.2007.03.007 |
| Duquette A. and McClintock J.B. and Amsler C.D., Pérez-Huerta A. and Milazzo M. and Hall-Spencer J.M. | Effects of ocean acidification on the shells of four Mediterranean gastropod species near a CO2 seep | 2017 | Marine Pollution Bulletin | 10.1016/j.marpolbul.2017.08.007 |
| Eckhardt J.-D. and Glasby G.P. and Puchelt H. and Berner Z. | Hydrothermal manganese crusts from Enarete and Palinuro seamounts in the Tyrrhenian Sea | 1997 | Marine Georesources and Geotechnology | 10.1080/10641199709379943 |
| Espa S. and Caramanna G. and Bouché V. | Field study and laboratory experiments of bubble plumes in shallow seas as analogues of sub-seabed CO2 leakages | 2010 | Applied Geochemistry | 10.1016/j.apgeochem.2010.02.002 |
| Esposito V. and Andaloro F. and Canese S. and Bortoluzzi G. and Bo M. and Di Bella M. and Italiano F. and Sabatino G. and Battaglia P. and Consoli P. and Giordano P. and Spagnoli F. and Cono V.L. and Yakimov M.M. and Scotti G. and Romeo T. | Exceptional discovery of a shallow-water hydrothermal site in the SW area of Basiluzzo islet (Aeolian archipelago, South Tyrrhenian Sea): An environment to preserve | 2018 | PLoS ONE | 10.1371/journal.pone.0190710 |
| Esposito V. and Auriemma R. and De Vittor C. and Relitti F. and Urbini L. and Kralj M. and Gambi M.C. | Structural and Functional Analyses of Motile Fauna Associated with Cystoseira brachycarpa along a Gradient of Ocean Acidification in a CO2-Vent System off Panarea (Aeolian Islands, Italy) | 2022 | Journal of Marine Science and Engineering | 10.3390/jmse10040451 |
| Esposito V. and Canese S. and Scotti G. and Bo M. and De Vittor C. and Andaloro F. and Romeo T. | Spiculosiphon oceana (foraminifera) and its affinity to intermediate stress conditions in the Panarea hydrothermal complex (Mediterranean Sea) | 2019 | Marine Biodiversity Records | 10.1186/s41200-019-0183-4 |
| Esposito V. and Giacobbe S. and Cosentino A. and Minerva C.S. and Romeo T. and Canese S. and Andaloro F. | Distribution and ecology of the tube-dweller Ampelisca ledoyeri (Amphipoda: Ampeliscidae) associated with the hydrothermal field off Panarea Island (Tyrrhenian Sea, Mediterranean) | 2015 | Marine Biodiversity | 10.1007/s12526-014-0285-5 |
| Etiope G. and Italiano F. and Fuda J.L. and Favali P. and Frugoni F. and Calcara M. and Smriglio G. and Gamberi F. and Marani M. | Deep submarine gas vents in the Aeolian offshore | 2000 | Physics and Chemistry of the Earth, Part B: Hydrology, Oceans and Atmosphere | 10.1016/S1464-1909(99)00115-X |
| Fanelli, E; Di Giacomo, S; Gambi, C; Bianchelli, S; Da Ros, Z; Tangherlini, M; Andaloro, F; Romeo, T; Corinaldesi, C; Danovaro, R | Effects of Local Acidification on Benthic Communities at Shallow Hydrothermal Vents of the Aeolian Islands (Southern Tyrrhenian, Mediterranean Sea) | 2022 | BIOLOGY-BASEL | 10.3390/biology11020321 |
| Fantazzini P. and Mengoli S. and Pasquini L. and Bortolotti V. and Brizi L. and Mariani M. and Di Giosia M. and Fermani S. and Capaccioni B. and Caroselli E. and Prada F. and Zaccanti F. and Levy O. and Dubinsky Z. and Kaandorp J.A. and Konglerd P. and Hammel J.U. and Dauphin Y. and Cuif J.-P. and Weaver J.C. and Fabricius K.E. and Wagermaier W. and Fratzl P. and Falini G. and Goffredo S. | Gains and losses of coral skeletal porosity changes with ocean acidification acclimation | 2015 | Nature Communications | 10.1038/ncomms8785 |
| Felden J. and Lichtschlag A. and WenzhÃ¶fer F. and De Beer D. and Feseker T. and Pop Ristova P. and De Lange G. and Boetius A. | Limitations of microbial hydrocarbon degradation at the Amon mud volcano (Nile deep-sea fan) | 2013 | Biogeosciences | 10.5194/bg-10-3269-2013 |
| Feseker T., Dählmann A. and Foucher J.-P. and Harmegnies F. | In-situ sediment temperature measurements and geochemical porewater data suggest highly dynamic fluid flow at Isis mud volcano, eastern Mediterranean Sea | 2009 | Marine Geology | 10.1016/j.margeo.2008.09.003 |
| Feseker, T; Brown and KR; Blanchet, C; Scholz, F; Nuzzo, M; Reitz, A; Schmidt, M; Hensen, C | Active mud volcanoes on the upper slope of the western Nile deep-sea fan-first results from the P362/2 cruise of R/V Poseidon | 2010 | Geo-Marine Letters | 10.1007/s00367-010-0192-0 |
| Fitzsimons M.F. and Dando P.R. and Hughes J.A. and Thiermann F. and Akoumianaki I. and Pratt S.M. | Submarine hydrothermal brine seeps off Milos, Greece: Observations and geochemistry | 1997 | Marine Chemistry | 10.1016/S0304-4203(97)00021-2 |
| Fontanier C. and Dissard D. and Ruffine L. and Mamo B. and Ponzevera E. and Pelleter E. and Baudin F. and Roubi A. and ChÃ©ron S. and Boissier A. and Gayet N. and Bermell-Fleury S. and Pitel M. and Guyader V. and Lesongeur F. and Savignac F. | Living (stained) deep-sea foraminifera from the Sea of Marmara: A preliminary study | 2018 | Deep-Sea Research Part II: Topical Studies in Oceanography | 10.1016/j.dsr2.2017.12.011 |
| Foo S.A. and Koweek D.A. and Munari M. and Gambi M.C. and Byrne M. and Caldeira K. | Responses of sea urchin larvae to field and laboratory acidification | 2020 | Science of the Total Environment | 10.1016/j.scitotenv.2020.138003 |
| Foutrakis P.M. and Anastasakis G. | The active submarine NW termination of the South Aegean Active Volcanic Arc: The Submarine Pausanias Volcanic Field | 2018 | Journal of Volcanology and Geothermal Research | 10.1016/j.jvolgeores.2018.05.008 |
| Franchi F. and Rovere M. and Gamberi F. and Rashed H. and Vaselli O. and Tassi F. | Authigenic minerals from the Paola Ridge (southern Tyrrhenian Sea): Evidences of episodic methane seepage | 2017 | Marine and Petroleum Geology | 10.1016/j.marpetgeo.2017.05.031 |
| Galindo-Zaldivar J. and Nieto L. and Woodside J. | Structural features of mud volcanoes and the fold system of the Mediterranean Ridge, South of Crete | 1996 | Marine Geology | 10.1016/0025-3227(96)00155-7 |
| Gallo A. and Boni R. and Buia M.C. and Monfrecola V. and Esposito M.C. and Tosti E. | Ocean acidification impact on ascidian Ciona robusta spermatozoa: New evidence for stress resilience | 2019 | Science of the Total Environment | 10.1016/j.scitotenv.2019.134100 |
| Gambi M.C. and Musco L. and Giangrande A. and Badalamenti F. and Micheli F. and Kroeker K.J. | Distribution and functional traits of polychaetes in a CO2 vent system: Winners and losers among closely related species | 2016 | Marine Ecology Progress Series | 10.3354/meps11727 |
| Garrard S.L. and Gambi M.C. and Scipione M.B. and Patti F.P. and Lorenti M. and Zupo V. and Paterson D.M. and Buia M.C. | Indirect effects may buffer negative responses of seagrass invertebrate communities to ocean acidification | 2014 | Journal of Experimental Marine Biology and Ecology | 10.1016/j.jembe.2014.07.011 |
| Gasperini L. and Polonia A. and Del Bianco F. and Etiope G. and Marinaro G. and Favali P. and Italiano F., Ã‡aÇ§atay M.N. | Gas seepage and seismogenic structures along the North Anatolian Fault in the eastern Sea of Marmara | 2012 | Geochemistry, Geophysics, Geosystems | 10.1029/2012GC004190 |
| Gaudron S.M. and Demoyencourt E. and Duperron S. | Reproductive traits of the cold-seep symbiotic mussel Idas modiolaeformis: Gametogenesis and larval biology | 2012 | Biological Bulletin | 10.1086/bblv222n1p6 |
| Gennari G. and Spezzaferri S. and Comas M.C., Rüggeberg A. and Lopez-Rodriguez C. and Pinheiro L.M. | Sedimentary sources of the mud-breccia and mud volcanic activity in the Western Alboran Basin | 2013 | Marine Geology | 10.1016/j.margeo.2013.04.002 |
| Giangrande A. and Gambi M.C. and Micheli F. and Kroeker K.J. | Fabriciidae (Annelida, Sabellida) from a naturally acidified coastal system (Italy) with description of two new species | 2014 | Journal of the Marine Biological Association of the United Kingdom | 10.1017/S0025315414000678 |
| Giangrande A. and Putignano M. and Licciano M. and Gambi M.C. | The Pandora's box: Morphological diversity within the genus Amphiglena Claparède, 1864 (Sabellidae, Annelida) in the Mediterranean Sea, with description of nine new species | 2021 | Zootaxa | 10.11646/zootaxa.4949.2.1 |
| Gill R.C.O. and Aparicio A. and El Azzouzi M. and Hernandez J. and Thirlwall M.F. and Bourgois J. and Marriner G.F. | Depleted arc volcanism in the Alboran Sea and shoshonitic volcanism in Morocco: Geochemical and isotopic constraints on Neogene tectonic processes | 2004 | Lithos | 10.1016/j.lithos.2004.07.002 |
| Giovannelli D., d'Errico G. and Manini E. and Yakimov M. and Vetriani C. | Diversity and phylogenetic analyses of bacteria from a shallow-water hydrothermal vent in Milos island (Greece) | 2013 | Frontiers in Microbiology | 10.3389/fmicb.2013.00184 |
| Giresse P. and Loncke L. and Huguen C. and Muller C. and Mascle J. | Nature and origin of sedimentary clasts associated with mud volcanoes in the Nile deep-sea fan Relationships with fluid venting | 2010 | Sedimentary Geology | 10.1016/j.sedgeo.2010.04.014 |
| Girnth A.-C. and Grunke S. and Lichtschlag A. and Felden J. and Knittel K. and Wenzhofer F. and De Beer D. and Boetius A. | A novel, mat-forming Thiomargarita population associated with a sulfidic fluid flow from a deep-sea mud volcano | 2011 | Environmental Microbiology | 10.1111/j.1462-2920.2010.02353.x |
| Gizzi F. and De Mas L. and Airi V. and Caroselli E. and Prada F. and Falini G. and Dubinsky Z. and Goffredo S. | Reproduction of an azooxanthellate coral is unaffected by ocean acidification | 2017 | Scientific Reports | 10.1038/s41598-017-13393-1 |
| Godelitsas A. and Price R.E. and Pichler T. and Amend J. and Gamaletsos P., Göttlicher J. | Amorphous As-sulfide precipitates from the shallow-water hydrothermal vents off Milos Island (Greece) | 2015 | Marine Chemistry | 10.1016/j.marchem.2015.09.004 |
| Goffredo S. and Prada F. and Caroselli E. and Capaccioni B. and Zaccanti F. and Pasquini L. and Fantazzini P. and Fermani S. and Reggi M. and Levy O. and Fabricius K.E. and Dubinsky Z. and Falini G. | Biomineralization control related to population density under ocean acidification | 2014 | Nature Climate Change | 10.1038/nclimate2241 |
| Gomez-Saez G.V. and Niggemann J. and Dittmar T. and Pohlabeln A.M. and Lang S.Q. and Noowong A. and Pichler T., WÃ¶rmer L., BÃ¼hring S.I. | Molecular evidence for abiotic sulfurization of dissolved organic matter in marine shallow hydrothermal systems | 2016 | Geochimica et Cosmochimica Acta | 10.1016/j.gca.2016.06.027 |
| Gontharet S. and Pierre C. and Blanc-Valleron M.-M. and Rouchy J.M. and Fouquet Y. and Bayon G. and Foucher J.P. and Woodside J. and Mascle J. | Nature and origin of diagenetic carbonate crusts and concretions from mud volcanoes and pockmarks of the Nile deep-sea fan (eastern Mediterranean Sea) | 2007 | Deep-Sea Research Part II: Topical Studies in Oceanography | 10.1016/j.dsr2.2007.04.007 |
| Gontharet S. and Stadnitskaia A. and Bouloubassi I. and Pierre C. and Damsté J.S.S. | Palaeo methane-seepage history traced by biomarker patterns in a carbonate crust, Nile deep-sea fan (Eastern Mediterranean Sea) | 2009 | Marine Geology | 10.1016/j.margeo.2008.11.006 |
| Goodwin, C; Rodolfo-Metalpa, R; Picton, B; Hall-Spencer and JM | Effects of ocean acidification on sponge communities | 2014 | Marine Ecology-An Evolutionary Perspective | 10.1111/maec.12093 |
| Gravili C. and Cozzoli F. and Gambi M.C. | Epiphytic hydroids on Posidonia oceanica seagrass meadows are winner organisms under future ocean acidification conditions: evidence from a CO2 vent system (Ischia Island, Italy) | 2021 | European Zoological Journal | 10.1080/24750263.2021.1899317 |
| Graziani S. and Beaubien S.E. and Bigi S. and Lombardi S. | Spatial and temporal pCO2 marine monitoring near Panarea Island (Italy) using multiple low-cost gaspro sensors | 2014 | Environmental Science and Technology | 10.1021/es500666u |
| Gros J. and Schmidt M. and Dale A.W. and Linke P. and Vielstädte L. and Bigalke N. and Haeckel M. and Wallmann K. and Sommer S. | Simulating and Quantifying Multiple Natural Subsea CO2 Seeps at Panarea Island (Aeolian Islands, Italy) as a Proxy for Potential Leakage from Subseabed Carbon Storage Sites | 2019 | Environmental Science and Technology | 10.1021/acs.est.9b02131 |
| Grunke S. and Felden J. and Lichtschlag A. and Girnth A.-C. and De Beer D. and Wenzhofer F. and Boetius A. | Niche differentiation among mat-forming, sulfide-oxidizing bacteria at cold seeps of the Nile Deep Sea Fan (Eastern Mediterranean Sea) | 2011 | Geobiology | 10.1111/j.1472-4669.2011.00281.x |
| Gugliandolo C. and Italiano F. and Maugeri T.L. and Inguaggiato S. and Caccamo D. and Amend J.P. | Submarine hydrothermal vents of the aeolian islands: Relationship between microbial communities and thermal fluids | 1999 | Geomicrobiology Journal | 10.1080/014904599270794 |
| Gugliandolo C. and Maugeri T.L. | Temporal variations in heterotrophic mesophilic bacteria from a marine shallow hydrothermal vent off the Island of Vulcano (Eolian Islands, Italy) | 1998 | Microbial Ecology | 10.1007/s002489900088 |
| Gugliandolo, C; Lentini, V; Bunk, B; Overmann, J; Italiano, F; Maugeri and TL | Changes in prokaryotic community composition accompanying a pronounced temperature shift of a shallow marine thermal brine pool (Panarea Island, Italy) | 2015 | EXTREMOPHILES | 10.1007/s00792-015-0737-2 |
| Guilini K. and Weber M. and De Beer D. and Schneider M. and Molari M. and Lott C. and Bodnar W. and Mascart T. and De Troch M. and Vanreusel A. | Response of Posidonia oceanica seagrass and its epibiont communities to ocean acidification | 2017 | PLoS ONE | 10.1371/journal.pone.0181531 |
| Haese R.R. and Hensen C. and De Lange G.J. | Pore water geochemistry of eastern Mediterranean mud volcanoes: Implications for fluid transport and fluid origin | 2006 | Marine Geology | 10.1016/j.margeo.2005.09.001 |
| Haese R.R. and Meile C. and Van Cappellen P. and De Lange G.J. | Carbon geochemistry of cold seeps: Methane fluxes and transformation in sediments from Kazan mud volcano, eastern Mediterranean Sea | 2003 | Earth and Planetary Science Letters | 10.1016/S0012-821X(03)00226-7 |
| Hahn, S; Rodolfo-Metalpa, R; Griesshaber, E; Schmahl and WW; Buhl, D; Hall-Spencer and JM; Baggini, C; Fehr and KT; Immenhauser, A | Marine bivalve shell geochemistry and ultrastructure from modern low pH environments: environmental effect versus experimental bias | 2012 | Biogeosciences | 10.5194/bg-9-1897-2012 |
| Hall-Spencer J.M. and Rodolfo-Metalpa R. and Martin S. and Ransome E. and Fine M. and Turner S.M. and Rowley S.J. and Tedesco D. and Buia M.-C. | Volcanic carbon dioxide vents show ecosystem effects of ocean acidification | 2008 | Nature | 10.1038/nature07051 |
| Heijs S.K. and Aloisi G. and Bouloubassi I. and Pancost R.D. and Pierre C. and Sinninghe DamstÃ© J.S. and Gottschal J.C. and Van Elsas J.D. and Forney L.J. | Microbial community structure in three deep-sea carbonate crusts | 2006 | Microbial Ecology | 10.1007/s00248-006-9099-8 |
| Heijs S.K. and Haese R.R. and Van Der Wielen P.W.J.J. and Forney L.J. and Van Elsas J.D. | Use of 16S rRNA gene based clone libraries to assess microbial communities potentially involved in anaerobic methane oxidation in a Mediterranean cold seep | 2007 | Microbial Ecology | 10.1007/s00248-006-9172-3 |
| Heijs S.K. and Sinninghe Damsté J.S. and Forney L.J. | Characterization of a deep-sea microbial mat from an active cold seep at the Milano mud volcano in the Eastern Mediterranean Sea | 2005 | FEMS Microbiology Ecology | 10.1016/j.femsec.2005.02.007 |
| Helene, O; Karine, O; Stephanie, D; Carla, S; Anne-Sophie, A; Clement, G; Livio, R | Geological and biological diversity of seeps in the Sea of Marmara | 2020 | Deep-Sea Research Part I: Oceanographic Research Papers | 10.1016/j.dsr.2020.103287 |
| Hilario A. and Comas M.C. and Azevedo L. and Pinheiro L. and Ivanov M.K. and Cunha M.R. | First record of a Vestimentifera (Polychaeta: Siboglinidae) from chemosynthetic habitats in the western Mediterranean Sea-Biogeographical implications and future exploration | 2011 | Deep-Sea Research Part I: Oceanographic Research Papers | 10.1016/j.dsr.2010.11.009 |
| Himmler T. and Brinkmann F. and Bohrmann G. and Peckmann J. | Corrosion patterns of seep-carbonates from the eastern Mediterranean Sea | 2011 | Terra Nova | 10.1111/j.1365-3121.2011.01000.x |
| Hodkinson R.A. and Cronan D.S. and Varnavas S. and Perissoratis C. | Regional geochemistry of sediments from the hellenic volcanic arc in regard to submarine hydrothermal activity | 1994 | Marine Georesources and Geotechnology | 10.1080/10641199409388257 |
| Holm N.G. | Possible biological origin of banded iron â€” Formations from hydrothermal solutions | 1987 | Origins of life and evolution of the biosphere | 10.1007/BF02386464 |
| Houghton J.L. and Gilhooly W.P. and III and Kafantaris F.-C.A. and Druschel G.K. and Lu G.-S. and Amend J.P. and Godelitsas A. and Fike D.A. | Spatially and temporally variable sulfur cycling in shallow-sea hydrothermal vents, Milos, Greece | 2019 | Marine Chemistry | 10.1016/j.marchem.2018.11.002 |
| Hubner A. and Rahders E. and Rahner S. and Halbach P. and Varnavas S.P. | Geochemistry of hydrothermally influenced sediments off Methana (western Hellenic volcanic arc) | 2004 | Chemie der Erde | 10.1016/j.chemer.2003.10.001 |
| Huguen C. and Foucher J.P. and Mascle J. and Ondréas H. and Thouement M. and Gontharet S. and Stadnitskaia A. and Pierre C. and Bayon G. and Loncke L. and Boetius A. and Bouloubassi I. and de Lange G. and Caprais J.C. and Fouquet Y. and Woodside J. and Dupré S. | Menes caldera, a highly active site of brine seepage in the Eastern Mediterranean sea: "In situ" observations from the NAUTINIL expedition (2003) | 2009 | Marine Geology | 10.1016/j.margeo.2009.02.005 |
| Iezzi G. and Caso C. and Ventura G. and Vallefuoco M. and Cavallo A. and Behrens H. and Mollo S. and Paltrinieri D. and Signanini P. and Vetere F. | First documented deep submarine explosive eruptions at the marsili seamount (tyrrhenian sea, italy): A case of historical volcanism in the mediterranean sea | 2014 | Gondwana Research | 10.1016/j.gr.2013.11.001 |
| Ingrassia M. and Martorelli E. and Bosman A. and Macelloni L. and Sposato A. and Chiocci F.L. | The Zannone Giant Pockmark: First evidence of a giant complex seeping structure in shallow-water, central Mediterranean Sea, Italy | 2015 | Marine Geology | 10.1016/j.margeo.2015.02.005 |
| Innangi S. and Passaro S. and Tonielli R. and Milano G. and Ventura G. and Tamburrino S. | Seafloor mapping using high-resolution multibeam backscatter: The Palinuro Seamount (Eastern Tyrrhenian Sea) | 2016 | Journal of Maps | 10.1080/17445647.2015.1071719 |
| Italiano F. and De Santis A. and Favali P. and Rainone M.L. and Rusi S. and Signanini P. | The Marsili Volcanic Seamount (Southern Tyrrhenian Sea): A potential offshore geothermal resource | 2014 | Energies | 10.3390/en7074068 |
| Italiano F. and Romano D. and Caruso C. and Longo M. and Corbo A. and Lazzaro G. | Magmatic Signature in Submarine Hydrothermal Fluids Vented Offshore Ventotene and Zannone Islands (Pontine Archipelago, Central Italy) | 2019 | Geofluids | 10.1155/2019/8759609 |
| Ivanov M.K. and Limonov A.F. and Van Weering Tj.C.E. | Comparative characteristics of the Black Sea and Mediterranean Ridge mud volcanoes | 1996 | Marine Geology | 10.1016/0025-3227(96)00165-X |
| Johnson and VR; Brownlee, C; Milazzo, M; Hall-Spencer and JM | Marine Microphytobenthic Assemblage Shift along a Natural Shallow-Water CO2 Gradient Subjected to Multiple Environmental Stressors | 2015 | Journal of Marine Science and Engineering | 10.3390/jmse3041425 |
| Johnson V.R. and Brownlee C. and Rickaby R.E.M. and Graziano M. and Milazzo M. and Hall-Spencer J.M. | Responses of marine benthic microalgae to elevated CO2 | 2013 | Marine Biology | 10.1007/s00227-011-1840-2 |
| Johnson V.R. and Russell B.D. and Fabricius K.E. and Brownlee C. and Hall-Spencer J.M. | Temperate and tropical brown macroalgae thrive, despite decalcification, along natural CO 2 gradients | 2012 | Global Change Biology | 10.1111/j.1365-2486.2012.02716.x |
| Jurado-Rodriguez M.J. and Martinez-Ruiz F. | Some clues about the Napoli and Milano mud volcanoes from an integrated log-core approach | 1998 | Proceedings of the Ocean Drilling Program: Scientific Results | 10.2973/odp.proc.sr.160.043.1998 |
| Kadar E. and Fisher A. and Stolpe B. and Harrison R.M. and Parello F. and Lead J. | Metallic nanoparticle enrichment at low temperature, shallow CO 2 seeps in Southern Italy | 2012 | Marine Chemistry | 10.1016/j.marchem.2012.07.001 |
| Kallscheuer N. and Wiegand S. and Boedeker C. and Peeters S.H. and Jogler M. and Heuer A. and Jetten M.S.M. and Rohde M. and Jogler C. | Caulifigura coniformis gen. nov., sp. nov., a novel member of the family Planctomycetaceae isolated from a red biofilm sampled in a hydrothermal area | 2020 | Antonie van Leeuwenhoek, International Journal of General and Molecular Microbiology | 10.1007/s10482-020-01439-w |
| Kallscheuer N. and Wiegand S. and Heuer A. and Rensink S. and Boersma A.S. and Jogler M. and Boedeker C. and Peeters S.H. and Rast P. and Jetten M.S.M. and Rohde M. and Jogler C. | Blastopirellula retiformator sp. nov. isolated from the shallow-sea hydrothermal vent system close to Panarea Island | 2020 | Antonie van Leeuwenhoek, International Journal of General and Molecular Microbiology | 10.1007/s10482-019-01377-2 |
| Kalogeropoulou V. and Keklikoglou K. and Lampadariou N. | Functional diversity patterns of abyssal nematodes in the Eastern Mediterranean: A comparison between cold seeps and typical deep sea sediments | 2015 | Journal of Sea Research | 10.1016/j.seares.2014.11.003 |
| Karuza A. and Celussi M. and Cibic T. and Del Negro P. and De Vittor C. | Virioplankton and bacterioplankton in a shallow CO 2-dominated hydrothermal vent (Panarea Island, Tyrrhenian Sea) | 2012 | Estuarine, Coastal and Shelf Science | 10.1016/j.ecss.2011.10.027 |
| Keller J. | Alkalibasalts from the Tyrrhenian sea Basin: Magmatic and geodynamic significance | 1981 | Bulletin Volcanologique | 10.1007/BF02600568 |
| Kerfahi D. and Hall-Spencer J.M. and Tripathi B.M. and Milazzo M. and Lee J. and Adams J.M. | Shallow Water Marine Sediment Bacterial Community Shifts Along a Natural CO2 Gradient in the Mediterranean Sea Off Vulcano, Italy | 2014 | Microbial Ecology | 10.1007/s00248-014-0368-7 |
| Kerrison P. and Hall-Spencer J.M. and Suggett D.J. and Hepburn L.J. and Steinke M. | Assessment of pH variability at a coastal CO2 vent for ocean acidification studies | 2011 | Estuarine, Coastal and Shelf Science | 10.1016/j.ecss.2011.05.025 |
| Khimasia A. and Renshaw C.E. and Price R.E. and Pichler T. | Hydrothermal flux and porewater geochemistry in Paleochori Bay, Milos, Greece | 2021 | Chemical Geology | 10.1016/j.chemgeo.2021.120188 |
| Kitidis V. and Laverock B. and McNeill L.C. and Beesley A. and Cummings D. and Tait K. and Osborn M.A. and Widdicombe S. | Impact of ocean acidification on benthic and water column ammonia oxidation | 2011 | Geophysical Research Letters | 10.1029/2011GL049095 |
| Kleindienst S. and Herbst F.-A. and Stagars M. and Von Netzer F. and Von Bergen M. and Seifert J. and Peplies J. and Amann R. and Musat F. and Lueders T. and Knittel K. | Diverse sulfate-reducing bacteria of the Desulfosarcina/Desulfococcus clade are the key alkane degraders at marine seeps | 2014 | ISME Journal | 10.1038/ismej.2014.51 |
| Kleindienst, S; Ramette, A; Amann, R; Knittel, K | Distribution and in situ abundance of sulfate-reducing bacteria in diverse marine hydrocarbon seep sediments | 2012 | Environmental Microbiology | 10.1111/j.1462-2920.2012.02832.x |
| Kordella S. and Christodoulou D. and Fakiris E. and Geraga M. and Kokkalas S. and Marinaro G. and Iatrou M. and Ferentinos G. and Papatheodorou G. | Gas seepage-induced features in the hypoxic/anoxic, shallow, marine environment of amfilochia bay, amvrakikos gulf (Western Greece) | 2021 | Geosciences (Switzerland) | 10.3390/geosciences11010027 |
| Kormas K.A. and Meziti A., Dählmann A. and De Lange G.J. and Lykousis V. | Characterization of methanogenic and prokaryotic assemblages based on mcrA and 16S rRNA gene diversity in sediments of the Kazan mud volcano (Mediterranean Sea) | 2008 | Geobiology | 10.1111/j.1472-4669.2008.00172.x |
| Kroeker K.J. and Gambi M.C. and Micheli F. | Community dynamics and ecosystem simplification in a high-CO2 ocean | 2013 | Proceedings of the National Academy of Sciences of the United States of America | 10.1073/pnas.1216464110 |
| Kroeker K.J. and Micheli F. and Gambi M.C. and Martz T.R. | Divergent ecosystem responses within a benthic marine community to ocean acidification | 2011 | Proceedings of the National Academy of Sciences of the United States of America | 10.1073/pnas.1107789108 |
| Laming S.R. and Duperron S. and Cunha M.R. and Gaudron S.M. | Settled, symbiotic, then sexually mature: Adaptive developmental anatomy in the deep-sea, chemosymbiotic mussel Idas modiolaeformis | 2014 | Marine Biology | 10.1007/s00227-014-2421-y |
| Lauritano C. and Ruocco M. and Dattolo E. and Buia M.C. and Silva J. and Santos R. and OlivÃ© I. and Costa M.M. and Procaccini G. | Response of key stress-related genes of the seagrass Posidonia oceanica in the vicinity of submarine volcanic vents | 2015 | Biogeosciences | 10.5194/bg-12-4185-2015 |
| Lazar C.S. and John Parkes R. and Cragg B.A., L'Haridon S. and Toffin L. | Methanogenic activity and diversity in the centre of the Amsterdam Mud Volcano, Eastern Mediterranean Sea | 2012 | FEMS Microbiology Ecology | 10.1111/j.1574-6941.2012.01375.x |
| Lazar C.S. and Parkes R.J. and Cragg B.A., L'Haridon S. and Toffin L. | Methanogenic diversity and activity in hypersaline sediments of the centre of the Napoli mud volcano, Eastern Mediterranean Sea | 2011 | Environmental Microbiology | 10.1111/j.1462-2920.2011.02425.x |
| Lazar C.S., L'Haridon S. and Pignet P. and Toffin L. | Archaeal populations in hypersaline sediments underlying orange microbial mats in the napoli mud volcano | 2011 | Applied and Environmental Microbiology | 10.1128/AEM.01296-10 |
| Lentini V. and Gugliandolo C. and Bunk B. and Overmann J. and Maugeri T.L. | Diversity of prokaryotic community at a shallow marine hydrothermal site elucidated by illumina sequencing technology | 2014 | Current Microbiology | 10.1007/s00284-014-0609-5 |
| L'Haridon S. and Chalopin M. and Colombo D. and Toffin L. | Methanococcoides vulcani sp. nov., a marine methylotrophic methanogen that uses betaine, choline and N,N-dimethylethanolamine for methanogenesis, isolated from a mud volcano, and emended description of the genus Methanococcoides | 2014 | International Journal of Systematic and Evolutionary Microbiology | 10.1099/ijs.0.058289-0 |
| Lietard C. and Pierre C. | High-resolution isotopic records (d18O and d13C) and cathodoluminescence study of lucinid shells from methane seeps of the Eastern Mediterranean | 2008 | Geo-Marine Letters | 10.1007/s00367-008-0100-z |
| Linares C. and Vidal M. and Canals M. and Kersting D.K. and Amblas D. and Aspillaga E. and Cebrián E. and Delgado-Huertas A., Díaz D. and Garrabou J. and Hereu B. and Navarro L. and Teixidó N. and Ballesteros E. | Persistent natural acidification drives major distribution shifts in marine benthic ecosystems | 2015 | Proceedings of the Royal Society B: Biological Sciences | 10.1098/rspb.2015.0587 |
| Liu Y. and Lu H. and Yin X. and Ruffine L., Ã‡aÄŸatay M.N. and Yang H. and Chen C. and He D. and Zhu Z. and Yalamaz B. | Interpretation of Late-Pleistocene/Holocene Transition in the Sea of Marmara From Geochemistry of Bulk Carbonates | 2019 | Geochemistry, Geophysics, Geosystems | 10.1029/2019GC008364 |
| Lodolo E. and Sanfilippo R. and Rajola G. and Canese S. and Andaloro F. and Montagna P. and Rosso A. and Macaluso D. and Geronimo I.D. and Caffau M. | The red coral deposits of the Graham Bank area: Constraints on the Holocene volcanic activity of the Sicilian Channel | 2017 | GeoResJ | 10.1016/j.grj.2017.04.003 |
| Loher M. and Marcon Y. and Pape T., Römer M. and Wintersteller P. and dos Santos Ferreira C. and Praeg D. and Torres M. and Sahling H. and Bohrmann G. | Seafloor sealing, doming, and collapse associated with gas seeps and authigenic carbonate structures at Venere mud volcano, Central Mediterranean | 2018 | Deep-Sea Research Part I: Oceanographic Research Papers | 10.1016/j.dsr.2018.04.006 |
| Loher, M; Ceramicola, S; Wintersteller, P; Meinecke, G; Sahling, H; Bohrmann, G | Mud Volcanism in a Canyon: Morphodynamic Evolution of the Active Venere Mud Volcano and Its Interplay With Squillace Canyon, Central Mediterranean | 2018 | Geochemistry, Geophysics, Geosystems | 10.1002/2017GC007166 |
| Lombardi C. and Gambi M.C. and Vasapollo C. and Taylor P. and Cocito S. | Skeletal alterations and polymorphism in a Mediterranean bryozoan at natural CO2 vents | 2011 | Zoomorphology | 10.1007/s00435-011-0127-y |
| Lombardi, C; Cocito, S; Gambi and MC; Cisterna, B; Flach, F; Taylor and PD; Keltie, K; Freer, A; Cusack, M | Effects of ocean acidification on growth, organic tissue and protein profile of the Mediterranean bryozoan Myriapora truncata | 2011 | Aquatic Biology | 10.3354/ab00376 |
| Loncke L. and Mascle J. | Mud volcanoes, gas chimneys, pockmarks and mounds in the Nile deep-sea fan (Eastern Mediterranean): Geophysical evidences | 2004 | Marine and Petroleum Geology | 10.1016/j.marpetgeo.2004.02.004 |
| Lopez-Rodri<U+00AD>guez C. and De Lange G.J. and Comas M. and Martínez-Ruiz F. and Nieto F. and Sapart C.J. and Mogollón J.M. | Recent, deep-sourced methane/mud discharge at the most active mud volcano in the western Mediterranean | 2019 | Marine Geology | 10.1016/j.margeo.2018.11.013 |
| Lopez-Rodri<U+00AD>guez C. and Stadnitskaia A. and De Lange G.J. and Martínez-Ruíz F. and Comas M. and Sinninghe Damsté J.S. | Origin of lipid biomarkers in mud volcanoes from the Alboran Sea, western Mediterranean | 2014 | Biogeosciences | 10.5194/bg-11-3187-2014 |
| Loreto M.F. and Italiano F. and Deponte D. and Facchin L. and Zgur F. | Mantle degassing on a near shore volcano, SE Tyrrhenian Sea | 2015 | Terra Nova | 10.1111/ter.12148 |
| Lu G.-S. and LaRowe D.E. and Fike D.A. and Druschel G.K. and Gilhooly W.P. and III and Price R.E. and Amend J.P. | Bioenergetic characterization of a shallow-sea hydrothermal vent system: Milos Island, Greece | 2020 | PLoS ONE | 10.1371/journal.pone.0234175 |
| Lucchi R.G. and Kidd R.B. | Sediment provenance and turbidity current processes at the Lametini Seamounts and Stromboli Canyon, SE Tyrrhenian Sea | 1998 | Geo-Marine Letters | 10.1007/s003670050063 |
| Lucey N.M. and Lombardi C. and Florio M. and DeMarchi L. and Nannini M. and Rundle S. and Gambi M.C. and Calosi P. | An in<U+00A0>situ assessment of local adaptation in a calcifying polychaete from a shallow CO2 vent system | 2016 | Evolutionary Applications | 10.1111/eva.12400 |
| Lucey N.M. and Lombardi C. and Florio M. and Rundle S.D. and Calosi P. and Gambi M.C. | A comparison of life-history traits in calcifying Spirorbinae polychaetes living along natural pH gradients | 2018 | Marine Ecology Progress Series | 10.3354/meps12453 |
| Lucila, M; Pomar and CA; Giuffre, G | Pico-, nano- and microplankton communities in hydrothermal marine coastal environments of the Eolian Islands (Panarea and Vulcano) in the Mediterranean Sea | 1996 | Journal of Plankton Research | 10.1093/plankt/18.5.715 |
| Lupton J. and De Ronde C. and Sprovieri M. and Baker E.T. and Bruno P.P. and Italiano F. and Walker S. and Faure K. and Leybourne M. and Britten K. and Greene R. | Active hydrothermal discharge on the submarine Aeolian Arc | 2011 | Journal of Geophysical Research: Solid Earth | 10.1029/2010JB007738 |
| Lykousis V. and Alexandri S. and Woodside J. and Nomikou P. and Perissoratis C. and Sakellariou D. and De Lange G. and Dahlmann A. and Casas D. and Rousakis G. and Ballas D. and Ioakim Chr. | New evidence of extensive active mud volcanism in the Anaximander mountains (Eastern Mediterranean): The "ATHINA" mud volcano | 2004 | Environmental Geology | 10.1007/s00254-004-1090-4 |
| Mandalakis M. and Gavriilidou A. and Polymenakou P.N. and Christakis C.A. and Nomikou P. and Medvecký M. and Kilias S.P. and Kentouri M. and Kotoulas G. and Magoulas A. | Microbial strains isolated from CO2-venting Kolumbo submarine volcano show enhanced co-tolerance to acidity and antibiotics | 2019 | Marine Environmental Research | 10.1016/j.marenvres.2019.01.002 |
| Manini E. and Luna G.M. and Corinaldesi C. and Zeppilli D. and Bortoluzzi G. and Caramanna G. and Raffa F. and Danovaro R. | Prokaryote diversity and virus abundance in shallow hydrothermal vents of the Mediterranean Sea (Panarea Island) and the Pacific Ocean (North Sulawesi-Indonesia) | 2008 | Microbial Ecology | 10.1007/s00248-007-9306-2 |
| Manno C. and Rumolo P. and Barra M., d'Albero S. and Basilone G. and Genovese S. and Mazzola S. and Bonanno A. | Condition of pteropod shells near a volcanic CO2 vent region | 2019 | Marine Environmental Research | 10.1016/j.marenvres.2018.11.003 |
| Marani M.P. and Gamberi F. and Savelli C. | Shallow-water polymetallic sulfide deposits in the Aeolian island arc | 1997 | Geology | 10.1130/0091-7613(1997)025<0815:SWPSDI>2.3.CO;2 |
| Marchini C. and Gizzi F. and Pondrelli T. and Moreddu L. and Marisaldi L. and Montori F. and Lazzari V. and Airi V. and Caroselli E. and Prada F. and Falini G. and Dubinsky Z. and Goffredo S. | Decreasing pH impairs sexual reproduction in a Mediterranean coral transplanted at a CO2 vent | 2021 | Limnology and Oceanography | 10.1002/lno.11937 |
| Margreth S. and Gennari G., Rüggeberg A. and Comas M.C. and Pinheiro L.M. and Spezzaferri S. | Growth and demise of cold-water coral ecosystems on mud volcanoes in the West Alboran Sea: The messages from the planktonic and benthic foraminifera | 2011 | Marine Geology | 10.1016/j.margeo.2011.02.006 |
| Marinaro G. and Etiope G. and Bue N.L. and Favali P. and Papatheodorou G. and Christodoulou D. and Furlan F. and Gasparoni F. and Ferentinos G. and Masson M. and Rolin J.-F. | Monitoring of a methane-seeping pockmark by cabled benthic observatory (Patras Gulf, Greece) | 2006 | Geo-Marine Letters | 10.1007/s00367-006-0040-4 |
| MartÃ­nez-Crego B. and Vizzini S. and Califano G. and Massa-Gallucci A. and Andolina C. and Gambi M.C. and Santos R. | Resistance of seagrass habitats to ocean acidification via altered interactions in a tri-trophic chain | 2020 | Scientific Reports | 10.1038/s41598-020-61753-1 |
| Martin S. and Rodolfo-Metalpa R. and Ransome E. and Rowley S. and Buia M.-C. and Gattuso J.-P. and Hall-Spencer J. | Effects of naturally acidified seawater on seagrass calcareous epibionts | 2008 | Biology Letters | 10.1098/rsbl.2008.0412 |
| Martorelli E. and Italiano F. and Ingrassia M. and Macelloni L. and Bosman A. and Conte A.M. and Beaubien S.E. and Graziani S. and Sposato A. and Chiocci F.L. | Evidence of a shallow water submarine hydrothermal field off Zannone Island from morphological and geochemical characterization: Implications for Tyrrhenian Sea Quaternary volcanism | 2016 | Journal of Geophysical Research: Solid Earth | 10.1002/2016JB013103 |
| Mascle G. and Lemoine M. and Mascle J. and Rehault J.P. and Tricart P. | Ophiolites and the oceanic crust: New evidence from the Tyrrhenian sea and the Western Alps | 1991 | Journal of Geodynamics | 10.1016/0264-3707(91)90036-E |
| Mastalerz V. and de Lange G.J., DÃ¤hlmann A. and Feseker T. | Active venting at the Isis mud volcano, offshore Egypt: Origin and migration of hydrocarbons | 2007 | Chemical Geology | 10.1016/j.chemgeo.2007.09.005 |
| Maugeri T.L. and Bianconi G. and Canganella F. and Danovaro R. and Gugliandolo C. and Italiano F. and Lentini V. and Manini E. and Nicolaus B. | Shallow hydrothermal vents in the southern Tyrrhenian Sea | 2010 | Chemistry and Ecology | 10.1080/02757541003693250 |
| Maugeri T.L. and Gugliandolo C. and Lentini V. | Diversity of prokaryotes at a shallow submarine vent of Panarea Island (Italy) by high-throughput sequencing | 2013 | AAPP Atti della Accademia Peloritana dei Pericolanti, Classe di Scienze Fisiche, Matematiche e Naturali | 10.1478/AAPP.912A1 |
| Maugeri T.L. and Lentini V. and Gugliandolo C. and Cousin S. and Stackebrandt E. | Microbial diversity at a hot, shallow-sea hydrothermal vent in the Southern Tyrrhenian Sea (Italy) | 2010 | Geomicrobiology Journal | 10.1080/01490450903451518 |
| Mecca S. and Casoli E. and Ardizzone G. and Gambi M.C. | Effects of ocean acidification on phenology and epiphytes of the seagrass Posidonia oceanica at two CO2 vent systems of Ischia (Italy) | 2020 | Mediterranean Marine Science | 10.12681/MMS.20795 |
| Megalovasilis P. | Partition geochemistry of hydrothermal precipitates from submarine hydrothermal fields in the Hellenic Volcanic Island Arc | 2014 | Geochemistry International | 10.1134/S0016702914110044 |
| Megalovasilis P. | Geochemistry of Hydrothermal Particles in Shallow Submarine Hydrothermal Vents on Milos Island, Aegean Sea East Mediterranean | 2020 | Geochemistry International | 10.1134/S001670292002007X |
| Megalovasilis P. | Hydrothermal Fluid Particle Geochemistry of Submarine Vents in Kos Island, Aegean Sea East Mediterranean | 2020 | Geochemistry International | 10.1134/S0016702920050067 |
| Merey S. and Longinos S.N. | Investigation of gas seepages in Thessaloniki mud volcano in the Mediterranean Sea | 2018 | Journal of Petroleum Science and Engineering | 10.1016/j.petrol.2018.05.014 |
| Meron D. and Buia M.-C. and Fine M. and Banin E. | Changes in Microbial Communities Associated with the Sea Anemone Anemonia viridis in a Natural pH Gradient | 2013 | Microbial Ecology | 10.1007/s00248-012-0127-6 |
| Meron D. and Rodolfo-Metalpa R. and Cunning R. and Baker A.C. and Fine M. and Banin E. | Changes in coral microbial communities in response to a natural pH gradient | 2012 | ISME Journal | 10.1038/ismej.2012.19 |
| Micallef A. and Spatola D. and Caracausi A. and Italiano F. and Barreca G., D'Amico S. and Petronio L. and Coren F. and Facchin L. and Blanos R. and Pavan A. and Paganini P. and Taviani M. | Active degassing across the Maltese Islands (Mediterranean Sea) and implications for its neotectonics | 2019 | Marine and Petroleum Geology | 10.1016/j.marpetgeo.2019.03.033 |
| Milazzo M. and Alessi C. and Quattrocchi F. and Chemello R., D'Agostaro R. and Gil J. and Vaccaro A.M. and Mirto S. and Gristina M. and Badalamenti F. | Biogenic habitat shifts under long-term ocean acidification show nonlinear community responses and unbalanced functions of associated invertebrates | 2019 | Science of the Total Environment | 10.1016/j.scitotenv.2019.02.391 |
| Milazzo M. and Rodolfo-Metalpa R. and Chan V.B.S. and Fine M. and Alessi C. and Thiyagarajan V. and Hall-Spencer J.M. and Chemello R. | Ocean acidification impairs vermetid reef recruitment | 2014 | Scientific Reports | 10.1038/srep04189 |
| Milazzo, M; Cattano, C; Alonzo and SH; Foggo, A; Gristina, M; Rodolfo-Metalpa, R; Sinopoli, M; Spatafora, D; Stiver and KA; Hall-Spencer and JM | Ocean acidification affects fish spawning but not paternity at CO2 seeps | 2016 | Proceedings of the Royal Society B: Biological Sciences | 10.1098/rspb.2016.1021 |
| Minniti M. and Bonavia F.F. | Copper-ore grade hydrothermal mineralization discovered in a seamount in the Tyrrhenian Sea (Mediterranean): Is the mineralization related to porphyry-coppers or to base metal lodes? | 1984 | Marine Geology | 10.1016/0025-3227(84)90097-5 |
| Mirasole A. and Badalamenti F. and Di Franco A. and Gambi M.C. and Teixidó N. | Boosted fish abundance associated with Posidonia oceanica meadows in temperate shallow CO2 vents | 2021 | Science of the Total Environment | 10.1016/j.scitotenv.2021.145438 |
| Mirasole A. and Gillanders B.M. and Reis-Santos P. and Grassa F. and Capasso G. and Scopelliti G. and Mazzola A. and Vizzini S. | The influence of high pCO2 on otolith shape, chemical and carbon isotope composition of six coastal fish species in a Mediterranean shallow CO2 vent | 2017 | Marine Biology | 10.1007/s00227-017-3221-y |
| Mirasole A. and Signa G. and Gianguzza P. and Bonaviri C. and Mazzola A. and Vizzini S. | Fish assemblages cope with ocean acidification in a shallow volcanic CO2 vent benefiting from an adjacent recovery area | 2020 | Marine Environmental Research | 10.1016/j.marenvres.2019.104851 |
| Mishra A.K. and Cabaço S. and de los Santos C.B. and Apostolaki E.T. and Vizzini S. and Santos R. | Long-term effects of elevated CO2 on the population dynamics of the seagrass Cymodocea nodosa: Evidence from volcanic seeps | 2021 | Marine Pollution Bulletin | 10.1016/j.marpolbul.2020.111824 |
| Mishra A.K. and Santos R. and Hall -Spencer J.M. | Elevated trace elements in sediments and seagrasses at CO2 seeps | 2020 | Marine Environmental Research | 10.1016/j.marenvres.2019.104810 |
| Molari M. and Guilini K. and Lins L. and Ramette A. and Vanreusel A. | CO2 leakage can cause loss of benthic biodiversity in submarine sands | 2019 | Marine Environmental Research | 10.1016/j.marenvres.2019.01.006 |
| Molari, M; Guilini, K; Lott, C; Weber, M; de Beer, D; Meyer, S; Ramette, A; Wegener, G; Wenzhofer, F; Martin, D; Cibic, T; De Vittor, C; Vanreusel, A; Boetius, A | CO2 leakage alters biogeochemical and ecological functions of submarine sands | 2018 | SCIENCE ADVANCES | 10.1126/sciadv.aao2040 |
| Morri C. and Blanchi C.N. and Cocito S. and Peirano A. and De Biase A.M. and Aliani S. and Pansini M. and Boyer M. and Ferdeghini F. and Pestarino M. and Dando P. | Biodiversity of marine sessile epifauna at an Aegean island subject to hydrothermal activity: Milos, eastern Mediterranean Sea | 1999 | Marine Biology | 10.1007/s002270050674 |
| Morri C. and Vafidis D. and Peirano A. and Chintiroglou C.C. and Bianchi C.N. | Anthozoa from a subtidal hydrothermal area of milos island (aegean sea), with notes on the construction potential of the scleractinian coral madracis pharensis | 2000 | Italian Journal of Zoology | 10.1080/11250000009356331 |
| Morten L. and Landini F. and Bocchi G. and Mottana A. and Brunfelt A.O. | FeMn crusts from the southern Tyrrhenian Sea | 1980 | Chemical Geology | 10.1016/0009-2541(80)90048-0 |
| Moskalenko V.N. | Volcanic-Sedimentary Deposits in the Tyrrhenian Abyssal Basin | 1992 | International Geology Review | 10.1080/00206819209465593 |
| Muller E.M. and Fine M. and Ritchie K.B. | The stable microbiome of inter and sub-tidal anemone species under increasing pCO2 | 2016 | Scientific Reports | 10.1038/srep37387 |
| Mutalipassi M. and Fink P. and Maibam C. and Porzio L. and Buia M.C. and Gambi M.C. and Patti F.P. and Scipione M.B. and Lorenti M. and Zupo V. | Ocean acidification alters the responses of invertebrates to wound-activated infochemicals produced by epiphytes of the seagrass Posidonia oceanica | 2020 | Journal of Experimental Marine Biology and Ecology | 10.1016/j.jembe.2020.151435 |
| Newcomb L.A. and Milazzo M. and Hall-Spencer J.M. and Carrington E. | Ocean acidification bends the mermaid's wineglass | 2015 | Biology Letters | 10.1098/rsbl.2014.1075 |
| Nikitas A. and Triantaphyllou M.V. and Rousakis G. and Panagiotopoulos I. and Pasadakis N. and Hatzianestis I. and Gogou A. | Pre-messinian deposits of the mediterranean ridge: Biostratigraphic and geochemical evidence from the olimpi mud volcano field | 2021 | Water (Switzerland) | 10.3390/w13101367 |
| Noe' S. and Bellavia C. and Calvo S. and Mazzola A. and Pirrotta M. and Sciandra M. and Vizzini S. and Tomasello A. | Resilience of the seagrass Posidonia oceanica following pulse-type disturbance | 2020 | Marine Environmental Research | 10.1016/j.marenvres.2020.105011 |
| Nogueira P. and Gambi M.C. and Vizzini S. and Califano G. and Tavares A.M. and Santos R. and Martínez-Crego B. | Altered epiphyte community and sea urchin diet in Posidonia oceanica meadows in the vicinity of volcanic CO2 vents | 2017 | Marine Environmental Research | 10.1016/j.marenvres.2017.04.002 |
| Nomikou P. and Carey S. and Papanikolaou D. and Croff Bell K. and Sakellariou D. and Alexandri M. and Bejelou K. | Submarine volcanoes of the Kolumbo volcanic zone NE of Santorini Caldera, Greece | 2012 | Global and Planetary Change | 10.1016/j.gloplacha.2012.01.001 |
| Nuzzo M. and Elvert M. and Schmidt M. and Scholz F. and Reitz A. and Hinrichs K.-U. and Hensen C. | Impact of hot fluid advection on hydrocarbon gas production and seepage in mud volcano sediments of thick Cenozoic deltas | 2012 | Earth and Planetary Science Letters | 10.1016/j.epsl.2012.05.009 |
| Olivé I. and Silva J. and Lauritano C. and Costa M.M. and Ruocco M. and Procaccini G. and Santos R. | Linking gene expression to productivity to unravel long-and short-term responses of seagrasses exposed to CO2 in volcanic vents | 2017 | Scientific Reports | 10.1038/srep42278 |
| Olu-Le Roy K. and Sibuet M. and Fiala-Médioni A. and Gofas S. and Salas C. and Mariotti A. and Foucher J.-P. and Woodside J. | Cold seep communities in the deep eastern Mediterranean Sea: Composition, symbiosis and spatial distribution on mud volcanoes | 2004 | Deep-Sea Research Part I: Oceanographic Research Papers | 10.1016/j.dsr.2004.07.004 |
| Omoregie E.O. and Mastalerz V. and De Lange G. and Straub K.L. and Kappler A., Røy H. and Stadnitskaia A. and Foucher J.-P. and Boetius A. | Biogeochemistry and community composition of iron- and sulfur-precipitating microbial mats at the Chefren mud volcano (Nile deep sea fan, eastern Mediterranean) | 2008 | Applied and Environmental Microbiology | 10.1128/AEM.01751-07 |
| Omoregie E.O. and Niemann H. and Mastalerz V. and de Lange G.J. and Stadnitskaia A. and Mascle J. and Foucher J.-P. and Boetius A. | Microbial methane oxidation and sulfate reduction at cold seeps of the deep Eastern Mediterranean Sea | 2009 | Marine Geology | 10.1016/j.margeo.2009.02.001 |
| Pachiadaki M.G. and Kallionaki A., Dählmann A. and De Lange G.J. and Kormas K.A. | Diversity and Spatial Distribution of Prokaryotic Communities Along A Sediment Vertical Profile of A Deep-Sea Mud Volcano | 2011 | Microbial Ecology | 10.1007/s00248-011-9855-2 |
| Pachiadaki M.G. and Lykousis V. and Stefanou E.G. and Kormas K.A. | Prokaryotic community structure and diversity in the sediments of an active submarine mud volcano (Kazan mud volcano, East Mediterranean Sea) | 2010 | FEMS Microbiology Ecology | 10.1111/j.1574-6941.2010.00857.x |
| Panagiotopoulos I.P. and Paraschos F. and Rousakis G. and Hatzianestis I. and Parinos C. and Morfis I. and Gogou A. | Assessment of the eruptive activity and identification of the mud breccia's source in the Olimpi mud volcano field, Eastern Mediterranean | 2020 | Deep-Sea Research Part II: Topical Studies in Oceanography | 10.1016/j.dsr2.2019.104701 |
| Pancost R.D and Hopmans E.C and Sinninghe Damsté J.S | Archaeal lipids in mediterranean cold seeps: Molecular proxies for anaerobic methane oxidation | 2001 | Geochimica et Cosmochimica Acta | 10.1016/S0016-7037(00)00562-7 |
| Panieri G. | The effect of shallow marine hydrothermal vent activity on benthic foraminifera (Aeolian arc, Tyrrhenian sea) | 2006 | Journal of Foraminiferal Research | 10.2113/36.1.3 |
| Panieri G. and Gamberi F. and Marani M. and Barbieri R. | Benthic foraminifera from a recent, shallow-water hydrothermal environment in the Aeolian Arc (Tyrrhenian Sea) | 2005 | Marine Geology | 10.1016/j.margeo.2005.04.002 |
| Pansini, M; Morri, C; Bianchi and CN | The sponge community of a subtidal area with hydrothermal vents: Miles Island, Aegean Sea | 2000 | Estuarine, Coastal and Shelf Science | 10.1006/ecss.2000.0674 |
| Pape T. and Kasten S. and Zabel M. and Bahr A. and Abegg F. and Hohnberg H.-J. and Bohrmann G. | Gas hydrates in shallow deposits of the Amsterdam mud volcano, Anaximander Mountains, northeastern Mediterranean Sea | 2010 | Geo-Marine Letters | 10.1007/s00367-010-0197-8 |
| Patoucheas P. and Koukousioura O. and Psarra S. and Aligizaki K. and Dimiza M.D. and Skampa E. and Michailidis I. and Nomikou P. and Triantaphyllou M.V. | Phytoplankton community structure changes during autumn and spring in response to environmental variables in Methana, Saronikos Gulf, Greece | 2021 | Environmental Science and Pollution Research | 10.1007/s11356-020-12272-z |
| Patwardhan S. and Foustoukos D.I. and Giovannelli D., YÃ¼cel M. and Vetriani C. | Ecological succession of sulfur-oxidizing epsilon- And gammaproteobacteria during colonization of a shallow-water gas vent | 2018 | Frontiers in Microbiology | 10.3389/fmicb.2018.02970 |
| Patwardhan S. and Vetriani C. | Varunaivibrio sulfuroxidans gen. Nov., sp. nov., a facultatively chemolithoautotrophic, mesophilic alphaproteobacterium from a shallow-water gas vent at Tor Caldara, Tyrrhenian Sea | 2016 | International Journal of Systematic and Evolutionary Microbiology | 10.1099/ijsem.0.001235 |
| Patwardhan, S; Smedile, F; Giovannelli, D; Vetriani, C | Metaproteogenomic Profiling of Chemosynthetic Microbial Biofilms Reveals Metabolic Flexibility During Colonization of a Shallow-Water Gas Vent | 2021 | Frontiers in Microbiology | 10.3389/fmicb.2021.638300 |
| Pena V. and Harvey B.P. and Agostini S. and Porzio L. and Milazzo M. and Horta P. and Le Gall L. and Hall-Spencer J.M. | Major loss of coralline algal diversity in response to ocean acidification | 2021 | Global Change Biology | 10.1111/gcb.15757 |
| Pérez-Rodriguez I. and Rawls M. and Katharine Coykendall D. and Foustoukos D.I. | Deferrisoma palaeochoriense sp. nov., a thermophilic, iron(III)-reducing bacterium from a shallow-water hydrothermal vent in the Mediterranean Sea | 2016 | International Journal of Systematic and Evolutionary Microbiology | 10.1099/ijsem.0.000798 |
| Peters M. and Strauss H. and Petersen S. and Kummer N.-A. and Thomazo C. | Hydrothermalism in the Tyrrhenian Sea: Inorganic and microbial sulfur cycling as revealed by geochemical and multiple sulfur isotope data | 2011 | Chemical Geology | 10.1016/j.chemgeo.2010.11.011 |
| Petersen S. and Monecke T. and Westhues A. and Hannington M.D. and Gemmell J.B. and Sharpe R. and Peters M. and Strauss H. and Lackschewitz K. and Augustin N. and Gibson H. and Kleeberg R. | Drilling shallow-water massive sulfides at the Palinuro volcanic complex, Aeolian Island arc, Italy | 2014 | Economic Geology | 10.2113/econgeo.109.8.2129 |
| Pettit and LR; Smart and CW; Hart and MB; Milazzo, M; Hall-Spencer and JM | Seaweed fails to prevent ocean acidification impact on foraminifera along a shallow-water CO2 gradient | 2015 | ECOLOGY AND EVOLUTION | 10.1002/ece3.1475 |
| Pierre C. and Bayon G. and Blanc-Valleron M.-M. and Mascle J. and Dupré S. | Authigenic carbonates related to active seepage of methane-rich hot brines at the Cheops mud volcano, Menes caldera (Nile deep-sea fan, eastern Mediterranean Sea) | 2014 | Geo-Marine Letters | 10.1007/s00367-014-0362-6 |
| Porzio L. and Buia M.C. and Ferretti V. and Lorenti M. and Rossi M. and Trifuoggi M. and Vergara A. and Arena C. | Photosynthesis and mineralogy of Jania rubens at low pH/high pCO2: A future perspective | 2018 | Science of the Total Environment | 10.1016/j.scitotenv.2018.02.065 |
| Porzio L. and Buia M.C. and Hall-Spencer J.M. | Effects of ocean acidification on macroalgal communities | 2011 | Journal of Experimental Marine Biology and Ecology | 10.1016/j.jembe.2011.02.011 |
| Porzio L. and Garrard S.L. and Buia M.C. | The effect of ocean acidification on early algal colonization stages at natural CO2 vents | 2013 | Marine Biology | 10.1007/s00227-013-2251-3 |
| Porzio, L; Buia and MC; Lorenti, M; Vitale, E; Amitrano, C; Arena, C | Ecophysiological response of Jania rubens (Corallinaceae) to ocean acidification | 2018 | RENDICONTI LINCEI-SCIENZE FISICHE E NATURALI | 10.1007/s12210-018-0719-2 |
| Prada F. and Caroselli E. and Mengoli S. and Brizi L. and Fantazzini P. and Capaccioni B. and Pasquini L. and Fabricius K.E. and Dubinsky Z. and Falini G. and Goffredo S. | Ocean warming and acidification synergistically increase coral mortality | 2017 | Scientific Reports | 10.1038/srep40842 |
| Price R.E. and LaRowe D.E. and Italiano F. and Savov I. and Pichler T. and Amend J.P. | Subsurface hydrothermal processes and the bioenergetics of chemolithoautotrophy at the shallow-sea vents off Panarea Island (Italy) | 2015 | Chemical Geology | 10.1016/j.chemgeo.2015.04.011 |
| Price R.E. and Lesniewski R. and Nitzsche K.S. and Meyerdierks A. and Saltikov C. and Pichler T. and Amend J.P. | Archaeal and bacterial diversity in an arsenic-rich shallow-Sea hydrothermal system undergoing phase separation | 2013 | Frontiers in Microbiology | 10.3389/fmicb.2013.00158 |
| Price R.E. and Savov I. and Planer-Friedrich B., BÃ¼hring S.I. and Amend J. and Pichler T. | Processes influencing extreme as enrichment in shallow-sea hydrothermal fluids of milos island, greece | 2013 | Chemical Geology | 10.1016/j.chemgeo.2012.06.007 |
| Rastelli E. and Corinaldesi C. and Dell'Anno A. and Tangherlini M. and Martorelli E. and Ingrassia M. and Chiocci F.L. and Lo Martire M. and Danovaro R. | High potential for temperate viruses to drive carbon cycling in chemoautotrophy-dominated shallow-water hydrothermal vents | 2017 | Environmental Microbiology | 10.1111/1462-2920.13890 |
| Ravaglioli C. and Lardicci C. and Pusceddu A. and Arpe E. and Bianchelli S. and Buschi E. and Bulleri F. | Ocean acidification alters meiobenthic assemblage composition and organic matter degradation rates in seagrass sediments | 2020 | Limnology and Oceanography | 10.1002/lno.11246 |
| Renzi M. and Romeo T. and Guerranti C. and Perra G. and Italiano F. and Focardi S.E. and Esposito V. and Andaloro F. | Temporal trends and matrix-dependent behaviors of trace elements closed to a geothermal hot-spot source (Aeolian Archipelago, Italy) | 2011 | Procedia Earth and Planetary Science | 10.1016/j.proeps.2011.11.003 |
| Ricevuto E. and Kroeker K.J. and Ferrigno F. and Micheli F. and Gambi M.C. | Spatio-temporal variability of polychaete colonization at volcanic CO2 vents indicates high tolerance to ocean acidification | 2014 | Marine Biology | 10.1007/s00227-014-2555-y |
| Ricevuto E. and Lanzoni I. and Fattorini D. and Regoli F. and Gambi M.C. | Arsenic speciation and susceptibility to oxidative stress in the fanworm Sabella spallanzanii (Gmelin) (Annelida, Sabellidae) under naturally acidified conditions: An in situ transplant experiment in a Mediterranean CO2 vent system | 2016 | Science of the Total Environment | 10.1016/j.scitotenv.2015.11.154 |
| Ricevuto E. and Vizzini S. and Gambi M.C. | Ocean acidification effects on stable isotope signatures and trophic interactions of polychaete consumers and organic matter sources at a CO2 shallow vent system | 2015 | Journal of Experimental Marine Biology and Ecology | 10.1016/j.jembe.2015.03.016 |
| Ristova P.P. and Wenzhöfer F. and Ramette A. and Felden J. and Boetius A. | Spatial scales of bacterial community diversity at cold seeps (Eastern Mediterranean Sea) | 2015 | ISME Journal | 10.1038/ismej.2014.217 |
| Ritt B. and Desbruyères D. and Caprais J.-C. and Gauthier O. and Ruffine L. and Buscail R. and Roy K.O.-L. and Sarrazin J. | Seep communities from two mud volcanoes in the deep eastern Mediterranean Sea: Faunal composition, spatial patterns and environmental control | 2012 | Marine Ecology Progress Series | 10.3354/meps09896 |
| Ritt B. and Duperron S. and Lorion J. and Sara Lazar C. and Sarrazin J. | Integrative study of a new cold-seep mussel (Mollusca: Bivalvia) associated with chemosynthetic symbionts in the Marmara Sea | 2012 | Deep-Sea Research Part I: Oceanographic Research Papers | 10.1016/j.dsr.2012.05.009 |
| Ritt B. and Pierre C. and Gauthier O. and WenzhÃ¶fer F. and Boetius A. and Sarrazin J. | Diversity and distribution of cold-seep fauna associated with different geological and environmental settings at mud volcanoes and pockmarks of the Nile Deep-Sea Fan | 2011 | Marine Biology | 10.1007/s00227-011-1679-6 |
| Ritt B. and Sarrazin J. and Caprais J.-C. and NoÃ«l P. and Gauthier O. and Pierre C. and Henry P. and DesbruyÃ¨res D. | First insights into the structure and environmental setting of cold-seep communities in the Marmara Sea | 2010 | Deep-Sea Research Part I: Oceanographic Research Papers | 10.1016/j.dsr.2010.05.011 |
| Rizzo and AL; Caracausi, A; Chavagnac, V; Nomikou, P; Polymenakou and PN; Mandalakis, M; Kotoulas, G; Magoulas, A; Castillo, A; Lampridou, D; Marusczak, N; Sonke and JE | Geochemistry of CO2-Rich Gases Venting From Submarine Volcanism: The Case of Kolumbo (Hellenic Volcanic Arc, Greece) | 2019 | Frontiers in Earth Science | 10.3389/feart.2019.00060 |
| Roberts H. and Price R. and Brombach C.-C. and Pichler T. | Mercury in the hydrothermal fluids and gases in Paleochori Bay, Milos, Greece | 2021 | Marine Chemistry | 10.1016/j.marchem.2021.103984 |
| Robertson A. | Mud volcanism on the Mediterranean Ridge: Initial results of Ocean Drilling Program Leg 160 | 1996 | Geology | 10.1130/0091-7613(1996)024<0239:MVOTMR>2.3.CO;2 |
| Robertson A.H.F. and Kopf A. | Origin of clasts and matrix within the Milano and Napoli mud volcanoes, Mediterranean Ridge accretionary complex | 1998 | Proceedings of the Ocean Drilling Program: Scientific Results | 10.2973/odp.proc.sr.160.044.1998 |
| Robin C. and Colantoni P. and Gennesseaux M. and Rehault J.P. | Vavilov seamount: A mildly alkaline Quaternary volcano in the Tyrrhenian Basin | 1987 | Marine Geology | 10.1016/0025-3227(87)90071-5 |
| Robinson C. | Plankton gross production and respiration in the shallow water hydrothermal systems of Milos, Aegean Sea | 2000 | Journal of Plankton Research | 10.1093/plankt/22.5.887 |
| Rodolfo-Metalpa R. and Lombardi C. and Cocito S. and Hall-Spencer J.M. and Gambi M.C. | Effects of ocean acidification and high temperatures on the bryozoan Myriapora truncata at natural CO2 vents | 2010 | Marine Ecology | 10.1111/j.1439-0485.2009.00354.x |
| Rodolfo-Metalpa R. and Montagna P. and Aliani S. and Borghini M. and Canese S. and Hall-Spencer J.M. and Foggo A. and Milazzo M. and Taviani M. and Houlbrèque F. | Calcification is not the Achilles' heel of cold-water corals in an acidifying ocean | 2015 | Global Change Biology | 10.1111/gcb.12867 |
| Rodolfo-Metalpa, R; Houlbreque, F; Tambutte, E; Boisson, F; Baggini, C; Patti and FP; Jeffree, R; Fine, M; Foggo, A; Gattuso and JP; Hall-Spencer and JM | Coral and mollusc resistance to ocean acidification adversely affected by warming | 2011 | Nature Climate Change | 10.1038/NCLIMATE1200 |
| Rodrigues C.F. and Duperron S. and Gaudron S.M. | First documented record of a living solemyid bivalve in a pockmark of the Nile Deep-sea Fan (eastern Mediterranean Sea) | 2011 | Marine Biodiversity Records | 10.1017/S175526721100008X |
| Rogelja M. and Cibic T. and Pennesi C. and De Vittor C. | Microphytobenthic community composition and primary production at gas and thermal vents in the Aeolian Islands (Tyrrhenian Sea, Italy) | 2016 | Marine Environmental Research | 10.1016/j.marenvres.2016.04.009 |
| Romano D. and Gattuso A. and Longo M. and Caruso C. and Lazzaro G. and Corbo A. and Italiano F. | Hazard Scenarios Related to Submarine Volcanic-Hydrothermal Activity and Advanced Monitoring Strategies: A Study Case from the Panarea Volcanic Group (Aeolian Islands, Italy) | 2019 | Geofluids | 10.1155/2019/8728720 |
| Romer M. and Sahling H. and Pape T. and dos Santos Ferreira C. and Wenzhöfer F. and Boetius A. and Bohrmann G. | Methane fluxes and carbonate deposits at a cold seep area of the Central Nile Deep Sea Fan, Eastern Mediterranean Sea | 2014 | Marine Geology | 10.1016/j.margeo.2013.10.011 |
| Rovere M. and Gamberi F. and Mercorella A. and Rashed H. and Gallerani A. and Leidi E. and Marani M. and Funari V. and Pini G.A. | Venting and seepage systems associated with mud volcanoes and mud diapirs in the southern Tyrrhenian Sea | 2014 | Marine Geology | 10.1016/j.margeo.2013.11.013 |
| Rovere M. and Rashed H. and Pecchioni E. and Mercorella A. and Ceregato A. and Leidi E. and Gamberi F. and Vaselli O. | Habitat mapping of cold seeps associated with authigenic mineralization (Paola Ridge, southern Tyrrhenian Sea): Combining seafloor backscatter with biogeochemistry signals | 2015 | Italian Journal of Geosciences | 10.3301/IJG.2014.15 |
| Ruff S.E. and Kuhfuss H. and Wegener G. and Lott C. and Ramette A. and Wiedling J. and Knittel K. and Weber M. | Methane seep in shallow-water permeable sediment harbors high diversity of anaerobic methanotrophic communities, Elba, Italy | 2016 | Frontiers in Microbiology | 10.3389/fmicb.2016.00374 |
| Rusch A. and Walpersdorf E. and deBeer D. and Gurrieri S. and Amend J.P. | Microbial communities near the oxic/anoxic interface in the hydrothermal system of Vulcano Island, Italy | 2005 | Chemical Geology | 10.1016/j.chemgeo.2005.07.026 |
| Rydell H.S. and Bonatti E. | Uranium in submarine metalliferous deposits | 1973 | Geochimica et Cosmochimica Acta | 10.1016/0016-7037(73)90265-2 |
| Salas C. and Woodside J. | Lucinoma kazani n. sp. (Mollusca: Bivalvia): Evidence of a living benthic community associated with a cold seep in the Eastern Mediterranean Sea | 2002 | Deep-Sea Research Part I: Oceanographic Research Papers | 10.1016/S0967-0637(02)00010-9 |
| San<e9> E. and Ingrassia M. and Martorelli E. and Chiocci F.L. | Amino acids in surface sediments of the Zannone Island shelf (Western Mediterranean Sea): Possible bioindicators of submarine hydrothermal activity | 2020 | Organic Geochemistry | 10.1016/j.orggeochem.2019.103960 |
| Saroni, A; Sciarra, A; Grassa, F; Eich, A; Weber, M; Lott, C; Ferretti, G; Ivaldi, R; Coltorti, M | Shallow submarine mud volcano in the northern Tyrrhenian sea, Italy | 2020 | Applied Geochemistry | 10.1016/j.apgeochem.2020.104722 |
| Sartoni G. and De Biasi A.M. | A survey of the marine algae of Milos Island, Greece | 1999 | Cryptogamie, Algologie | 10.1016/S0181-1568(99)80019-1 |
| Sautkin A. and Talukder A.R. and Comas M.C. and Soto J.I. and Alekseev A. | Mud volcanoes in the Alboran Sea: Evidence from micropaleontological and geophysical data | 2003 | Marine Geology | 10.1016/S0025-3227(02)00691-6 |
| Savelli C. and Gasparotto G. | Calc-alkaline magmatism and rifting of the deep-water volcano of Marsili (Aeolian back-arc, Tyrrhenian Sea) | 1994 | Marine Geology | 10.1016/0025-3227(94)90145-7 |
| Savelli C. and Marani M. and Gamberi F. | Geochemistry of metalliferous, hydrothermal deposits in the Aeolian arc (Tyrrhenian Sea) | 1999 | Journal of Volcanology and Geothermal Research | 10.1016/S0377-0273(99)00007-4 |
| Scartazza A. and Moscatello S. and Gavrichkova O. and Buia M.C. and Lauteri M. and Battistelli A. and Lorenti M. and Garrard S.L. and Calfapietra C. and Brugnoli E. | Carbon and nitrogen allocation strategy in Posidonia oceanica is altered by seawater acidification | 2017 | Science of the Total Environment | 10.1016/j.scitotenv.2017.06.084 |
| Schmidt M. and Linke P. and Sommer S. and Esser D. and Cherednichenko S. | Natural CO2 seeps offshore panarea: A test site for subsea CO2 leak detection technology | 2014 | Marine Technology Society Journal | 10.4031/MTSJ.49.1.3 |
| Scholz F. and Hensen C. and De Lange G.J. and Haeckel M. and Liebetrau V. and Meixner A. and Reitz A. and Romer R.L. | Lithium isotope geochemistry of marine pore waters - Insights from cold seep fluids | 2010 | Geochimica et Cosmochimica Acta | 10.1016/j.gca.2010.03.026 |
| Schulz H.-M. and Emeis K.-C. and Volkmann N. | Organic carbon provenance and maturity in the mud breccia from the Napoli mud volcano: Indicators of origin and burial depth | 1997 | Earth and Planetary Science Letters | 10.1016/s0012-821x(97)00013-7 |
| Sciarra A. and Saroni A. and Etiope G. and Coltorti M. and Mazzarini F. and Lott C. and Grassa F. and Italiano F. | Shallow submarine seep of abiotic methane from serpentinized peridotite off the Island of Elba, Italy | 2019 | Applied Geochemistry | 10.1016/j.apgeochem.2018.10.025 |
| Scipione M.B. | On the presence of the Mediterranean endemic Microdeutopus sporadhi Myers, 1969 (Crustacea: Amphipoda: Aoridae) in the Gulf of Naples (Italy) with a review on its distribution and ecology | 2013 | Mediterranean Marine Science | 10.12681/mms.650 |
| Sciutteri V. and Smedile F. and Vizzini S. and Mazzola A. and Vetriani C. | Microbial Biofilms Along a Geochemical Gradient at the Shallow-Water Hydrothermal System of Vulcano Island, Mediterranean Sea | 2022 | Frontiers in Microbiology | 10.3389/fmicb.2022.840205 |
| Sedwick P. and StÃ¼ben D. | Chemistry of shallow submarine warm springs in an arc-volcanic setting: Vulcano Island, Aeolian Archipelago, Italy | 1996 | Marine Chemistry | 10.1016/0304-4203(96)00020-5 |
| Sievert S.M. and Kuever J. and Muyzer G. | Identification of 16s ribosomal DNA-defined bacterial populations at a shallow submarine hydrothermal vent near Milos island (Greece) | 2000 | Applied and Environmental Microbiology | 10.1128/AEM.66.7.3102-3109.2000 |
| Sisma-Ventura G. and Bialik O.M. and Makovsky Y. and Rahav E. and Ozer T. and Kanari M. and Marmen S. and Belkin N. and Guy-Haim T. and Antler G. and Herut B. and Rubin-Blum M. | Cold seeps alter the near-bottom biogeochemistry in the ultraoligotrophic Southeastern Mediterranean Sea | 2022 | Deep-Sea Research Part I: Oceanographic Research Papers | 10.1016/j.dsr.2022.103744 |
| Smith P.A. and Cronan D.S. | The geochemistry of metalliferous sediments and waters associated with shallow submarine hydrothermal activity (Santorini, Aegean Sea) | 1983 | Chemical Geology | 10.1016/0009-2541(83)90017-7 |
| Southward E.C. and Andersen A.C. and Hourdez S. | Lamellibrachia anaximandri n. Sp., a new vestimentiferan tubeworm (Annelida) from the Mediterranean, with notes on frenulate tubeworms from the same habitat | 2011 | Zoosystema | 10.5252/z2011n3a1 |
| Spatafora D. and Quattrocchi F. and Cattano C. and Badalamenti F. and Milazzo M. | Nest guarding behaviour of a temperate wrasse differs between sites off Mediterranean CO2 seeps | 2021 | Science of the Total Environment | 10.1016/j.scitotenv.2021.149376 |
| Stanulla R. and Pohl T., Müller C. and Engel J. and Hoyer M. and Merkel B. | Structural and mineralogical study of active and inactive hydrothermal fluid discharges in Panarea, Italy | 2017 | Environmental Earth Sciences | 10.1007/s12665-017-6714-6 |
| Stathopoulou P.M. and Savvides A.L. and Karagouni A.D. and Hatzinikolaou D.G. | Unraveling the lipolytic activity of thermophilic bacteria isolated from a volcanic environment | 2013 | BioMed Research International | 10.1155/2013/703130 |
| Suggett D.J. and Hall-Spencer J.M. and Rodolfo-Metalpa R. and Boatman T.G. and Payton R. and Tye Pettay D. and Johnson V.R. and Warner M.E. and Lawson T. | Sea anemones may thrive in a high CO 2 world | 2012 | Global Change Biology | 10.1111/j.1365-2486.2012.02767.x |
| Szafranski K.M. and Deschamps P. and Cunha M.R. and Gaudron S.M. and Duperron S. | Colonization of plant substrates at hydrothermal vents and cold seeps in the northeast Atlantic and Mediterranean and occurrence of symbiont-related bacteria | 2015 | Frontiers in Microbiology | 10.3389/fmicb.2015.00162 |
| Talas E. and Duman M., Küçüksezgin F. and Brennan M.L. and Raineault N.A. | Sedimentology and geochemistry of mud volcanoes in the Anaximander Mountain Region from the Eastern Mediterranean Sea | 2015 | Marine Pollution Bulletin | 10.1016/j.marpolbul.2015.04.042 |
| Tamborrino L. and Himmler T. and Elvert M. and Conti S. and Gualtieri A.F. and Fontana D. and Bohrmann G. | Formation of tubular carbonate conduits at Athina mud volcano, eastern Mediterranean Sea | 2019 | Marine and Petroleum Geology | 10.1016/j.marpetgeo.2019.05.003 |
| Tamburrino S. and Vallefuoco M. and Ventura G. and Insinga D.D. and Sprovieri M. and Tiepolo M. and Passaro S. | The proximal marine record of the Marsili Seamount in the last 7ka (Southern Tyrrhenian Sea, Italy): Implications for the active processes in the Tyrrhenian Sea back-arc | 2015 | Global and Planetary Change | 10.1016/j.gloplacha.2015.07.005 |
| Tangherlini M. and Corinaldesi C. and Ape F. and Greco S. and Romeo T. and Andaloro F. and Danovaro R. | Ocean acidification induces changes in virusâ€“host relationships in mediterranean benthic ecosystems | 2021 | Microorganisms | 10.3390/microorganisms9040769 |
| Tassi F. and Vaselli O. and Papazachos C.B. and Giannini L. and Chiodini G. and Vougioukalakis G.E. and Karagianni E. and Vamvakaris D. and Panagiotopoulos D. | Geochemical and isotopic changes in the fumarolic and submerged gas discharges during the 2011-2012 unrest at Santorini caldera (Greece) | 2013 | Bulletin of Volcanology | 10.1007/s00445-013-0711-8 |
| Taviani, M; Angeletti, L; Ceregato, A; Foglini, F; Froglia, C; Trincardi, F | The Gela Basin pockmark field in the strait of Sicily (Mediterranean Sea): chemosymbiotic faunal and carbonate signatures of postglacial to modern cold seepage | 2013 | Biogeosciences | 10.5194/bg-10-4653-2013 |
| Taylor J.D. and Ellis R. and Milazzo M. and Hall-Spencer J.M. and Cunliffe M. | Intertidal epilithic bacteria diversity changes along a naturally occurring carbon dioxide and pH gradient | 2014 | FEMS Microbiology Ecology | 10.1111/1574-6941.12368 |
| Teichert B.M.A. and Chevalier N. and Gussone N. and Bayon G. and Ponzevera E. and Ruffine L. and Strauss H. | Sulfate-dependent anaerobic oxidation of methane at a highly dynamic bubbling site in the Eastern Sea of Marmara (Ã‡inarcik Basin) | 2018 | Deep-Sea Research Part II: Topical Studies in Oceanography | 10.1016/j.dsr2.2017.11.014 |
| TeixidÃ³ N. and Caroselli E. and Alliouane S. and Ceccarelli C. and Comeau S. and Gattuso J.-P. and Fici P. and Micheli F. and Mirasole A. and Monismith S.G. and Munari M. and Palumbi S.R. and Sheets E. and Urbini L. and De Vittor C. and Goffredo S. and Gambi M.C. | Ocean acidification causes variable trait-shifts in a coral species | 2020 | Global Change Biology | 10.1111/gcb.15372 |
| Thiel V., Hügler M. and Blümel M. and Baumann H.I., Gärtner A. and Schmaljohann R. and Strauss H. and Garbe-Schönberg D. and Petersen S. and Cowart D.A. and Fisher C.R. and Imhoff J.F. | Widespread occurrence of two carbon fixation pathways in tubeworm endosymbionts: Lessons from hydrothermal vent associated tubeworms from the mediterranean sea | 2012 | Frontiers in Microbiology | 10.3389/fmicb.2012.00423 |
| Thiermann F. and Akoumianaki I. and Hughes J.A. and Giere O. | Benthic fauna of a shallow-water gaseohydrothermal vent area in the Aegean Sea (Milos, Greece) | 1997 | Marine Biology | 10.1007/s002270050078 |
| Treude T. and Kiel S. and Linke P. and Peckmann J. and Goedert J.L. | Elasmobranch egg capsules associated with modern and ancient cold seeps: A nursery for marine deep-water predators | 2011 | Marine Ecology Progress Series | 10.3354/meps09305 |
| Triantaphyllou M.V. and Baumann K.-H. and Karatsolis B.-T. and Dimiza M.D. and Psarra S. and Skampa E. and Patoucheas P. and Vollmar N.M. and Koukousioura O. and Katsigera A. and Krasakopoulou E. and Nomikou P. | Coccolithophore community response along a natural CO2 gradient off Methana (SW Saronikos Gulf, Greece, NE Mediterranean) | 2018 | PLoS ONE | 10.1371/journal.pone.0200012 |
| Tribollet A. and Grange J.S. and Parra H. and Rodolfo-Metalpa R. and Carreiro-Silva M. | Limited Carbonate Dissolution by Boring Microflora at Two Volcanically Acidified Temperate Sites: Ischia (Italy, Mediterranean Sea) and Faial (Azores, NE Atlantic Ocean) | 2018 | Global Biogeochemical Cycles | 10.1002/2016GB005575 |
| Trotter J. and Montagna P. and McCulloch M. and Silenzi S. and Reynaud S. and Mortimer G. and Martin S. and Ferrier-Pagès C. and Gattuso J.-P. and Rodolfo-Metalpa R. | Quantifying the pH 'vital effect' in the temperate zooxanthellate coral Cladocora caespitosa: Validation of the boron seawater pH proxy | 2011 | Earth and Planetary Science Letters | 10.1016/j.epsl.2011.01.030 |
| Trua T. and Serri G. and Marani M. and Renzulli A. and Gamberi F. | Volcanological and petrological evolution of Marsili seamount (southern Tyrrhenian Sea) | 2002 | Journal of Volcanology and Geothermal Research | 10.1016/S0377-0273(01)00300-6 |
| Trua T. and Serri G. and Marani M.P. | Geochemical features and geodynamic significance of the southern Tyrrhenian backarc basin | 2007 | Special Paper of the Geological Society of America | 10.1130/2007.2418(11) |
| Tsabaris C. and Patiris D.L. and Pappa F.K. and Alexakis S. and Michalopoulos P. | Preliminary investigation of olimpi field, Mediterranean Sea, using in-situ and laboratory radio-tracing methods | 2020 | Deep-Sea Research Part II: Topical Studies in Oceanography | 10.1016/j.dsr2.2019.104689 |
| Tudino T. and Bortoluzzi G. and Aliani S. | Shallow-water gaseohydrothermal plume studies after massive eruption at Panarea, Aeolian Islands, Italy | 2014 | Journal of Marine Systems | 10.1016/j.jmarsys.2013.10.001 |
| Turner L.M. and Ricevuto E. and Massa-Gallucci A. and Gambi M.-C. and Calosi P. | Energy metabolism and cellular homeostasis trade-offs provide the basis for a new type of sensitivity to ocean acidification in a marine polychaete at a high-CO2 vent: Adenylate and phosphagen energy pools versus carbonic anhydrase | 2015 | Journal of Experimental Biology | 10.1242/jeb.117705 |
| Ugur A. and Miquel J.-C. and Fowler S.W. and Appleby P. | Radiometric dating of sediment cores from a hydrothermal vent zone off Milos Island in the Aegean Sea | 2003 | Science of the Total Environment | 10.1016/S0048-9697(02)00542-9 |
| Valvassori G. and Benedetti M. and Regoli F. and Gambi M.C. | Antioxidant efficiency of platynereis spp.(Annelida, Nereididae) under different ph conditions at a CO2 Vent's System | 2019 | Journal of Marine Biology | 10.1155/2019/8415916 |
| Van Der Meer R. | Grading in mud volcanic breccia from the mediterranean ridge | 1996 | Marine Geology | 10.1016/0025-3227(95)00159-X |
| Varnavas S.P. | Submarine hydrothermal metallogenesis associated with the collision of two plates: The Southern Aegean Sea region | 1989 | Geochimica et Cosmochimica Acta | 10.1016/0016-7037(89)90271-8 |
| Varnavas S.P. and Cronan D.S. | Arsenic, antimony and bismuth in sediments and waters from the Santorini hydrothermal field, Greece | 1988 | Chemical Geology | 10.1016/0009-2541(88)90135-0 |
| Varnavas S.P. and Cronan D.S. | Hydrothermal metallogenic processes off the islands of Nisiros and Kos in the Hellenic Volcanic Arc | 1991 | Marine Geology | 10.1016/0025-3227(91)90086-J |
| Varnavas S.P. and Cronan D.S. | Submarine hydrothermal activity off Santorini and Milos in the Central Hellenic Volcanic Arc: A synthesis | 2005 | Chemical Geology | 10.1016/j.chemgeo.2005.07.013 |
| Varnavas S.P. and Panagiotaras D. and Megalovasilis P. and Dando P. and Alliani S. and Meloni R. | Compositional characterization of suspended particulate matter in Hellenic Volcanic Arc hydrothermal centres | 2000 | Physics and Chemistry of the Earth, Part B: Hydrology, Oceans and Atmosphere | 10.1016/S1464-1909(99)00113-6 |
| Varnavas S.P. and Papaioannou J. and Catani J. | A hydrothermal manganese deposit from the Eratosthenes Seamount, Eastern Mediterranean Sea | 1988 | Marine Geology | 10.1016/0025-3227(88)90027-8 |
| Ventura P. and Jarrold M.D. and Merle P.-L. and Barnay-Verdier S. and Zamoum T. and Rodolfo-Metalpa R. and Calosi P. and Furla P. | Resilience to ocean acidification: Decreased carbonic anhydrase activity in sea anemones under high pCO2 conditions | 2016 | Marine Ecology Progress Series | 10.3354/meps11916 |
| Vizzini S. and Apostolaki E.T. and Ricevuto E. and Polymenakou P. and Mazzola A. | Plant and sediment properties in seagrass meadows from two Mediterranean CO2 vents: Implications for carbon storage capacity of acidified oceans | 2019 | Marine Environmental Research | 10.1016/j.marenvres.2019.03.001 |
| Vizzini S. and Di Leonardo R. and Costa V. and Tramati C.D. and Luzzu F. and Mazzola A. | Trace element bias in the use of CO2 vents as analogues for low pH environments: Implications for contamination levels in acidified oceans | 2013 | Estuarine, Coastal and Shelf Science | 10.1016/j.ecss.2013.09.015 |
| Vizzini S. and Tomasello A. and Di Maida G. and Pirrotta M. and Mazzola A. and Calvo S. | Effect of explosive shallow hydrothermal vents on <U+0394>13C and growth performance in the seagrass Posidonia oceanica | 2010 | Journal of Ecology | 10.1111/j.1365-2745.2010.01730.x |
| Volgin A.V. and Woodside J.M. | Sidescan sonar images of mud volcanoes from the Mediterranean Ridge: Possible causes of variations in backscatter intensity | 1996 | Marine Geology | 10.1016/0025-3227(95)00152-2 |
| Voltattorni N. and Sciarra A. and Caramanna G. and Cinti D. and Pizzino L. and Quattrocchi F. | Gas geochemistry of natural analogues for the studies of geological CO2 sequestration | 2009 | Applied Geochemistry | 10.1016/j.apgeochem.2009.04.026 |
| Voudouris P. and Kati M. and Magganas A. and Keith M. and Valsami-Jones E. and Haase K. and Klemd R. and Nestmeyer M. | Arsenian pyrite and cinnabar from active submarine nearshore vents, paleochori bay, milos island, Greece | 2021 | Minerals | 10.3390/min11010014 |
| WÃ¤ge J. and Rotchell J.M. and Gambi M.-C. and Hardege J.D. | Target gene expression studies on Platynereis dumerilii and Platynereis cfr massiliensis at the shallow CO2 vents off Ischia, Italy | 2018 | Estuarine, Coastal and Shelf Science | 10.1016/j.ecss.2017.11.012 |
| Wage J. and Valvassori G. and Hardege J.D. and Schulze A. and Gambi M.C. | The sibling polychaetes Platynereis dumerilii and Platynereis massiliensis in the Mediterranean Sea: are phylogeographic patterns related to exposure to ocean acidification? | 2017 | Marine Biology | 10.1007/s00227-017-3222-x |
| Wakefield and SJ; OSullivan and GM | The inorganic geochemistry of a Mediterranean Ridge mud breccia | 1996 | Marine Geology | 10.1016/0025-3227(95)00161-1 |
| Wall M. and Prada F. and Fietzke J. and Caroselli E. and Dubinsky Z. and Brizi L. and Fantazzini P. and Franzellitti S. and Mass T. and Montagna P. and Falini G. and Goffredo S. | Linking Internal Carbonate Chemistry Regulation and Calcification in Corals Growing at a Mediterranean CO2 Vent | 2019 | Frontiers in Marine Science | 10.3389/fmars.2019.00699 |
| Werne J.P. and Haese R.R. and Zitter T. and Aloisi G. and Bouloubassi I. and Heijs S. and Fiala-Médioni A. and Pancost R.D. and Sinninghe Damsté J.S. and de Lange G. and Forney L.J. and Gottschal J.C. and Foucher J.-P. and Mascle J. and Woodside J. | Life at cold seeps: A synthesis of biogeochemical and ecological data from Kazan mud volcano, eastern Mediterranean Sea | 2004 | Chemical Geology | 10.1016/j.chemgeo.2003.12.031 |
| Werne J.P. and Sinninghe Damsté J.S. | Mixed sources contribute to the molecular isotopic signature of methane-rich mud breccia sediments of Kazan mud volcano (eastern Mediterranean) | 2005 | Organic Geochemistry | 10.1016/j.orggeochem.2004.07.013 |
| Witkowski C.R. and van der Meer M.T.J. and Smit N.T. and Sinninghe DamstÃ© J.S. and Schouten S. | Testing algal-based pCO2 proxies at a modern CO2 seep (Vulcano, Italy) | 2020 | Scientific Reports | 10.1038/s41598-020-67483-8 |
| Wu S.-F. and You C.-F. and Lin Y.-P. and Valsami-Jones E. and Baltatzis E. | New boron isotopic evidence for sedimentary and magmatic fluid influence in the shallow hydrothermal vent system of Milos Island (Aegean Sea, Greece) | 2016 | Journal of Volcanology and Geothermal Research | 10.1016/j.jvolgeores.2015.11.013 |
| Wu S.-F. and You C.-F. and Wang B.-S. and Valsami-Jones E. and Baltatzis E. | Two-cells phase separation in shallow submarine hydrothermal system at Milos Island, Greece: Boron isotopic evidence | 2011 | Geophysical Research Letters | 10.1029/2011GL047409 |
| Wulf S. and Keller J. and Satow C. and Gertisser R. and Kraml M. and Grant K.M. and Appelt O. and Vakhrameeva P. and Koutsodendris A. and Hardiman M. and Schulz H. and Pross J. | Advancing Santorini's tephrostratigraphy: New glass geochemical data and improved marine-terrestrial tephra correlations for the past ~360 kyrs | 2020 | Earth-Science Reviews | 10.1016/j.earscirev.2019.102964 |
| Yakimov M.M. and Giuliano L. and Cappello S. and Denaro R. and Golyshin P.N. | Microbial community of a hydrothermal mud vent underneath the deep-sea anoxic brine lake Urania (Eastern Mediterranean) | 2007 | Origins of Life and Evolution of the Biosphere | 10.1007/s11084-006-9021-x |
| Yamasaki, H; Grzelak, K; Sorensen and MV; Neuhaus, B; George and KH | Echinoderes pterus sp n. showing a geographically and bathymetrically wide distribution pattern on seamounts and on the deep-sea floor in the Arctic Ocean, Atlantic Ocean, and the Mediterranean Sea (Kinorhyncha, Cyclorhagida) | 2018 | ZOOKEYS | 10.3897/zookeys.771.25534 |
| Yücel M. and Sievert S.M. and Vetriani C. and Foustoukos D.I. and Giovannelli D. and Le Bris N. | Eco-geochemical dynamics of a shallow-water hydrothermal vent system at Milos Island, Aegean Sea (Eastern Mediterranean) | 2013 | Chemical Geology | 10.1016/j.chemgeo.2013.07.020 |
| Zeppilli D. and Canals M. and Danovaro R. | Pockmarks enhance deep-sea benthic biodiversity: A case study in the western Mediterranean Sea | 2012 | Diversity and Distributions | 10.1111/j.1472-4642.2011.00859.x |
| Zeppilli D. and Mea M. and Corinaldesi C. and Danovaro R. | Mud volcanoes in the Mediterranean Sea are hot spots of exclusive meiobenthic species | 2011 | Progress in Oceanography | 10.1016/j.pocean.2011.01.001 |
| Zimmermann J. and Lott C. and Weber M. and Ramette A. and Bright M. and Dubilier N. and Petersen J.M. | Dual symbiosis with co-occurring sulfur-oxidizing symbionts in vestimentiferan tubeworms from a Mediterranean hydrothermal vent | 2014 | Environmental Microbiology | 10.1111/1462-2920.12427 |
| Zitter and TAC; Henry, P; Aloisi, G; Delaygue, G; Cagatay and MN; de Lepinay and BM; Al-Samir, M; Fornacciari, F; Tesmer, M; Pekdeger, A; Wallmann, K; Lericolais, G | Cold seeps along the main Marmara Fault in the Sea of Marmara (Turkey) | 2008 | Deep-Sea Research Part I: Oceanographic Research Papers | 10.1016/j.dsr.2008.01.002 |
| Zitter T.A.C. and Huguen C. and Woodside J.M. | Geology of mud volcanoes in the eastern Mediterranean from combined sidescan sonar and submersible surveys | 2005 | Deep-Sea Research Part I: Oceanographic Research Papers | 10.1016/j.dsr.2004.10.005 |
| Ziveri P. and Passaro M. and Incarbona A. and Milazzo M. and Rodolfo-Metalpa R. and Hall-Spencer J.M. | Decline in coccolithophore diversity and impact on coccolith morphogenesis along a natural CO2 gradient | 2014 | Biological Bulletin | 10.1086/BBLv226n3p282 |
| Zvi-Kedem, T; Shemesh, E; Tchernov, D; Rubin-Blum, M | The worm affair: fidelity and environmental adaptation in symbiont species that co-occur in vestimentiferan tubeworms | 2021 | Environmental Microbiology Reports | 10.1111/1758-2229.12994 |
