# Supplementary material for: Volcanic-associated ecosystems of the Mediterranean Sea: a systematic map and an interactive tool to support their conservation
Source: PeerJ. 2023 Mar 29;11:e15162. doi: 10.7717/peerj.15162 (PMC10066691; doi:10.7717/peerj.15162)
Supplement: Supplemental Information 4 — The list of the sites includes name, latitude, longitude, average depth reported (in m) and type of site extracted from the scientific items included in the Systematic Map database. [file peerj-11-15162-s004.docx]

**Table S4:**

**Complete list of the sites included in the Systematic Map.**

The list of the sites includes name, latitude, longitude, average depth reported (in m) and type of site extracted from the scientific items included in the Systematic Map database.

| # | Region | Site | Latitude | Longitude | Average depth (m) | Site Type |
| --- | --- | --- | --- | --- | --- | --- |
| 1 | Aegean Arc | Kallisti Limnes | 36.45343 | 25.40279 | 235 | Volcanic Area |
| 2 | Aegean Arc | Kolumbo | 36.525 | 25.4833 | 495 | Volcanic Area |
| 3 | Aegean Arc | Kos | 36.85 | 27.25 | 4 | Volcanic Area |
| 4 | Aegean Arc | Methana | 37.63333 | 23.36667 | 6 | Hydrothermal Vents |
| 5 | Aegean Arc | Milos | 36.6667 | 24.5167 | 32 | Hydrothermal Vents |
| 6 | Aegean Arc | Santorini | 36.4333 | 25.4 | 1 | Volcanic Area |
| 7 | Aegean Arc | Yali | 36.66278 | 27.12519 | 2 | Hydrothermal Vents |
| 8 | Aegean Arc |  | 36.05 | 27.18667 | 806 | Seamount |
| 9 | Aeolian Arc | Alcione | 39.29717 | 15.29878 | 1760 | Seamount |
| 10 | Aeolian Arc | Alicudi | 38.53729 | 14.33322 | 146 | Hydrothermal Vents |
| 11 | Aeolian Arc | Basiluzzo | 38.67081 | 15.12607 | 118 | Hydrothermal Vents |
| 12 | Aeolian Arc | Basiluzzo-Fe | 38.67253 | 15.12849 | 140 | Hydrothermal Vents |
| 13 | Aeolian Arc | Black Point | 38.63972 | 15.10778 | 23 | Hydrothermal Vents |
| 14 | Aeolian Arc | Enarete | 38.64233 | 14.00036 | 1660 | Seamount |
| 15 | Aeolian Arc | Eolo | 38.56311 | 14.15862 | 1370 | Volcanic Area |
| 16 | Aeolian Arc | Glabro | 39.51302 | 15.16295 | 1200 | Seamount |
| 17 | Aeolian Arc | Hot Lake | 38.64053 | 15.11003 | 22 | Hydrothermal Vents |
| 18 | Aeolian Arc | La Calcara | 38.6459 | 15.0753 | 20 | Hydrothermal Vents |
| 19 | Aeolian Arc | Lametini | 38.98293 | 15.33344 | 1820 | Seamount |
| 20 | Aeolian Arc | Lipari | 38.46743 | 14.95398 | 3 | Hydrothermal Vents |
| 21 | Aeolian Arc | Magnaghi | 39.87558 | 11.75812 | 3140 | Seamount |
| 22 | Aeolian Arc | Marsili | 39.26 | 14.378 | 3180 | Volcanic Area |
| 23 | Aeolian Arc | Palinuro | 39.55 | 14.7 | 1580 | Volcanic Area |
| 24 | Aeolian Arc | Panarea | 38.6583 | 15.1 | 23 | Hydrothermal Vents |
| 25 | Aeolian Arc | Panarea-Basiluzzo | 38.65088 | 15.10255 | 40 | Hydrothermal Vents |
| 26 | Aeolian Arc | Panarea-Black Point | 38.63972 | 15.10778 | 22 | Hydrothermal Vents |
| 27 | Aeolian Arc | Panarea-Bottaro | 38.63769 | 15.1092 | 10 | Hydrothermal Vents |
| 28 | Aeolian Arc | Panarea-Campo 7 | 38.63838 | 15.10647 | 20 | Hydrothermal Vents |
| 29 | Aeolian Arc | Panarea-HotCold Vents | 38.639714 | 15.079353 | 11 | Hydrothermal Vents |
| 30 | Aeolian Arc | Panarea-Secca dei Pesci | 38.59467 | 15.11562 | 40 | Hydrothermal Vents |
| 31 | Aeolian Arc | PCTD3 | 38.64281 | 15.08649 | 45 | Hydrothermal Vents |
| 32 | Aeolian Arc | Secca del Capo | 38.63833 | 14.85 | 122 | Hydrothermal Vents |
| 33 | Aeolian Arc | Sisifo | 38.78828 | 13.85064 | 2020 | Seamount |
| 34 | Aeolian Arc | Smoking Land | 38.65694 | 15.1 | 75 | Hydrothermal Vents |
| 35 | Aeolian Arc | Stromboli | 38.789 | 15.213 | 1 | Hydrothermal Vents |
| 36 | Aeolian Arc | Vavilov | 39.86683 | 12.59346 | 3150 | Seamount |
| 37 | Aeolian Arc | Vulcano | 38.4 | 15 | 1 | Hydrothermal Vents |
| 38 | Alboran Basin | Alboran ridge | 35.46257 | -3.96417 | 445 | Volcanic Area |
| 39 | Alboran Basin | Carmen | 35.72183 | -4.73433 | 809 | Mud Volcano |
| 40 | Alboran Basin | Ceuta-south | 35.58952 | -4.68537 | 685 | Pockmarks |
| 41 | Alboran Basin | Crow's foot | 35.56867 | -4.71112 | 572 | Pockmarks |
| 42 | Alboran Basin | Dhaka | 35.42383 | -4.5315 | 370 | Mud Volcano |
| 43 | Alboran Basin | Granada | 35.56363 | -4.61945 | 615 | Mud Volcano |
| 44 | Alboran Basin | Kalinin | 36.05 | -4.93167 | 908 | Mud Volcano |
| 45 | Alboran Basin | Marrakech | 35.62733 | -4.49618 | 1086 | Mud Volcano |
| 46 | Alboran Basin | Maya | 35.45183 | -4.619 | 410 | Mud Volcano |
| 47 | Alboran Basin | Mulhacene | 35.4071 | -4.56877 | 365 | Mud Volcano |
| 48 | Alboran Basin | Perejil | 36.10183 | -4.88467 | 845 | Mud Volcano |
| 49 | Alboran Basin | Schneider's Heart | 36.00467 | -4.9595 | 924 | Mud Volcano |
| 50 | Alboran Basin | Yusurf ridge | 35.91578 | -2.10518 | 445 | Volcanic Area |
| 51 | Anaximander Mountains | Amsterdam | 35.33098 | 30.27547 | 2030 | Cold Seeps |
| 52 | Anaximander Mountains | Anaximander | 35.4797 | 30.0066 | 1800 | Mud Volcano |
| 53 | Anaximander Mountains | Anaximenes | 35.4307 | 30.1642 | 700 | Seamount |
| 54 | Anaximander Mountains | Athina | 35.3877 | 30.21018 | 1800 | Mud Volcano |
| 55 | Anaximander Mountains | Faulted Ridge | 35.45167 | 30.46667 | 1300 | Cold Seeps |
| 56 | Anaximander Mountains | Kazan | 35.67565 | 30.51171 | 1700 | Mud Volcano |
| 57 | Anaximander Mountains | Kula | 35.67565 | 30.51171 | 1630 | Mud Volcano |
| 58 | Anaximander Mountains | Saint Ouen l'Aumone | 35.37096 | 30.89252 | 2000 | Mud Volcano |
| 59 | Anaximander Mountains | Thessaloniki | 35.47672 | 30.25188 | 1264 | Mud Volcano |
| 60 | Anaximander Mountains | Tuzlukush | 35.37148 | 30.78667 | 2000 | Mud Volcano |
| 61 | Calabrian Arc | Athena | 37.43754 | 16.75144 | 2400 | Mud Volcano |
| 62 | Calabrian Arc | Bortoluzzi | 37.88361 | 16.28056 | 1400 | Mud Volcano |
| 63 | Calabrian Arc | Capo Vaticano | 38.69167 | 15.72389 | 800 | Seamount |
| 64 | Calabrian Arc | Catanzaro | 38.81743 | 16.67069 | 150 | Mud Volcano |
| 65 | Calabrian Arc | Cerere | 38.20205 | 17.06855 | 1600 | Mud Volcano |
| 66 | Calabrian Arc | Giunone | 38.04006 | 17.42075 | 1500 | Mud Volcano |
| 67 | Calabrian Arc | Madonna dello Ionio | 38.18377 | 16.88679 | 1700 | Mud Volcano |
| 68 | Calabrian Arc | Minerva | 38.79145 | 17.6801 | 1750 | Mud Volcano |
| 69 | Calabrian Arc | Pythagoras | 37.75763 | 17.15731 | 1987 | Mud Volcano |
| 70 | Calabrian Arc | Sartori | 38.17366 | 17.45921 | 1850 | Mud Volcano |
| 71 | Calabrian Arc | Venere | 38.57296 | 17.17007 | 1600 | Mud Volcano |
| 72 | Central Mediterranean | Korbous | 36.81667 | 10.56667 | 1 | Volcanic Area |
| 73 | Eastern Mediterranean | Amfilochia PM | 38.87811 | 21.17246 | 36 | Pockmarks |
| 74 | Eastern Mediterranean | Eratosthenes | 33.71517 | 32.59582 | 1300 | Pockmarks |
| 75 | Eastern Mediterranean | no-name | 31.65 | 33.45 | 550 | Cold Seeps |
| 76 | Eastern Mediterranean | Palmachim | 32.16667 | 34.16667 | 1036 | Volcanic Area |
| 77 | Eastern Mediterranean | Patras Gulf | 38.2125 | 21.7125 | 42 | Pockmarks |
| 78 | Eastern Mediterranean | Pockmarks Area | 32.53336 | 30.35209 | 1692 | Pockmarks |
| 79 | Eastern Mediterranean | Urania | 35.23083 | 21.47333 | 3700 | Hydrothermal Vents |
| 80 | Elba Pianosa Ridge | Scoglio d'Affrica | 42.37543 | 10.07669 | 12 | Mud Volcano |
| 81 | Gulf of Lion | Aude canyon | 42.69783 | 3.792333 | 288 | Pockmarks |
| 82 | Marmara Sea | Central Basin | 40.855 | 28.17 | 1120 | Cold Seeps |
| 83 | Marmara Sea | Central High | 40.86417 | 28.58361 | 326 | Cold Seeps |
| 84 | Marmara Sea | Cinarcik Basin | 40.78306 | 29.10639 | 1120 | Cold Seeps |
| 85 | Marmara Sea | Darica | 40.71915 | 29.41136 | 101 | Cold Seeps |
| 86 | Marmara Sea | Tekirdag Basin | 40.80306 | 27.62944 | 1110 | Cold Seeps |
| 87 | Marmara Sea | Western High | 40.80701 | 27.76491 | 592 | Cold Seeps |
| 88 | Mediterranean Ridge | Cobblestone area | 35.73666 | 20.79474 | 3800 | Pockmarks |
| 89 | Mediterranean Ridge | Jaén | 33.87328 | 24.84177 | 2239 | Mud Volcano |
| 90 | Mediterranean Ridge | Milano | 33.73147 | 22.7779 | 1952 | Mud Volcano |
| 91 | Mediterranean Ridge | Milford Haven | 33.77703 | 24.60846 | 1850 | Mud Volcano |
| 92 | Mediterranean Ridge | Moscow | 33.66667 | 24.5 | 1838 | Mud Volcano |
| 93 | Mediterranean Ridge | Nezhinka | 33.72409 | 24.45413 | 1915 | Mud Volcano |
| 94 | Mediterranean Ridge | Prometheus 2 | 33.83409 | 24.43182 | 1996 | Volcanic Area |
| 95 | Mediterranean Ridge | Stvor | 33.67985 | 24.60181 | 2000 | Mud Volcano |
| 96 | Mediterranean Ridge | Toronto | 33.43744 | 24.73282 | 2123 | Mud Volcano |
| 97 | Gulf of Naples | Ischia | 40.7306 | 13.95 | 3 | Hydrothermal Vents |
| 98 | Gulf of Naples | Ischia-Castello | 40.73208 | 13.96403 | 3 | Hydrothermal Vents |
| 99 | Gulf of Naples | Ischia-Grotta del Mago | 40.71163 | 13.96418 | 5 | Hydrothermal Vents |
| 100 | Gulf of Naples | Naples Bay | 40.61143 | 14.7627 | 172 | Hydrothermal Vents |
| 101 | Gulf of Naples | Phlegrean Fields | 40.80629 | 14.12183 | 21 | Volcanic Area |
| 102 | Gulf of Naples | Secca delle Fumose | 40.82306 | 14.0875 | 15 | Hydrothermal Vents |
| 103 | Gulf of Naples | Torre del Greco | 40.7497 | 14.37617 | 100 | Volcanic Area |
| 104 | Nile Deep-Sea Fan | Amon | 32.3708 | 31.7139 | 1120 | Mud Volcano |
| 105 | Nile Deep-Sea Fan | Caldera | 32.07831 | 28.61051 | 2953 | Mud Volcano |
| 106 | Nile Deep-Sea Fan | Central | 32.54066 | 30.55564 | 1907 | Mud Volcano |
| 107 | Nile Deep-Sea Fan | Central Pockmark | 32.89058 | 30.58672 | 1690 | Pockmarks |
| 108 | Nile Deep-Sea Fan | Central Province | 32.53306 | 30.3525 | 1800 | Pockmarks |
| 109 | Nile Deep-Sea Fan | Central Zone | 32.53472 | 30.35639 | 1687 | Cold Seeps |
| 110 | Nile Deep-Sea Fan | Central Zone 2A | 32.53472 | 30.35639 | 1694 | Cold Seeps |
| 111 | Nile Deep-Sea Fan | Central-lower | 32.54066 | 30.55564 | 1907 | Mud Volcano |
| 112 | Nile Deep-Sea Fan | Central-Middle | 32.54066 | 30.55564 | 1907 | Mud Volcano |
| 113 | Nile Deep-Sea Fan | Chefren | 32.07831 | 28.61051 | 3020 | Mud Volcano |
| 114 | Nile Deep-Sea Fan | Cheops | 32.14142 | 28.1619 | 3020 | Mud Volcano |
| 115 | Nile Deep-Sea Fan | Chephren | 32.07831 | 28.61051 | 3020 | Mud Volcano |
| 116 | Nile Deep-Sea Fan | Giza | 31.63609 | 29.73316 | 700 | Mud Volcano |
| 117 | Nile Deep-Sea Fan | Isis | 32.4174 | 31.4721 | 992 | Mud Volcano |
| 118 | Nile Deep-Sea Fan | Menitites | 32.14067 | 28.13967 | 3010 | Mud Volcano |
| 119 | Nile Deep-Sea Fan | Mykerinos | 32.07831 | 28.61051 | 2953 | Mud Volcano |
| 120 | Nile Deep-Sea Fan | North Alex | 31.93968 | 30.08077 | 507 | Mud Volcano |
| 121 | Nile Deep-Sea Fan | Osiris | 32.32833 | 31.59167 | 747 | Mud Volcano |
| 122 | Nile Deep-Sea Fan | Small MV1 | 32.14133 | 28.161 | 3012 | Mud Volcano |
| 123 | Nile Deep-Sea Fan | Small MV2 | 32.14033 | 28.15883 | 3005 | Mud Volcano |
| 124 | Nile Deep-Sea Fan | South Chephren | 32.1065 | 28.17783 | 3000 | Mud Volcano |
| 125 | Olimpi mud field | Bergamo | 33.72961 | 24.74335 | 1986 | Mud Volcano |
| 126 | Olimpi mud field | Gelendzhik | 33.90268 | 24.2689 | 1700 | Mud Volcano |
| 127 | Olimpi mud field | Heraklion | 33.93777 | 24.11403 | 1722 | Mud Volcano |
| 128 | Olimpi mud field | Leipzig | 33.77539 | 24.64418 | 1912 | Mud Volcano |
| 129 | Olimpi mud field | Maidstone | 33.6329 | 24.6785 | 2000 | Mud Volcano |
| 130 | Olimpi mud field | Monza | 33.72814 | 24.7216 | 2021 | Mud Volcano |
| 131 | Olimpi mud field | Napoli | 33.72909 | 24.68238 | 1940 | Mud Volcano |
| 132 | Olimpi mud field | Olimpi | 33.75 | 24.75 | 1850 | Mud Volcano |
| 133 | Paola Basin | Paola Ridge | 36.16909 | 15.36203 | 727 | Cold Seeps |
| 134 | Pontine Archipelago | La Botte | 40.8526 | 13.07062 | 212 | Volcanic Area |
| 135 | Pontine Archipelago | Palmarola | 40.93417 | 12.8573 | 24 | Volcanic Area |
| 136 | Pontine Archipelago | Ponza | 40.89709 | 12.95935 | 26 | Volcanic Area |
| 137 | Pontine Archipelago | Zannone | 40.97122 | 13.0558 | 150 | Volcanic Area |
| 138 | Sicily Channel | Gela | 36.76062 | 14.00251 | 900 | Pockmarks |
| 139 | Sicily Channel | Gozo Channel | 36.00866 | 14.32072 | 180 | Volcanic Area |
| 140 | Sicily Channel | Graham bank | 37.17835 | 12.70836 | 172 | Volcanic Area |
| 141 | Sicily Channel | Malta Plateau | 36.55 | 14.4667 | 153 | Mud Volcano |
| 142 | Sicily Channel | Nerita bank | 37.24154 | 12.90184 | 200 | Volcanic Area |
| 143 | Sicily Channel | Pantelleria | 37.43741 | 12.17774 | 35 | Volcanic Area |
| 144 | Sicily Channel | Pantelleria (1891) | 36.84486 | 11.89122 | 350 | Volcanic Area |
| 145 | Sicily Channel | Sicily channel | 36.57111 | 14.5025 | 141 | Mud Volcano |
| 146 | Sicily Channel | Sicily-south | 36.57111 | 14.5025 | 141 | Mud Volcano |
| 147 | Sicily Channel | Terrible bank | 37.12212 | 12.82505 | 45 | Pockmarks |
| 148 | Tyrrhenian | Pomonte | 42.7438 | 10.11864 | 12 | Cold Seeps |
| 149 | Tyrrhenian | Presidiana | 38.03824 | 14.0324 | 1 | Volcanic Area |
| 150 | Tyrrhenian | Tor Caldara | 41.48583 | 12.58972 | 3 | Hydrothermal Vents |
| 151 | Tyrrhenian | Vercelli | 40.98672 | 10.77094 | 2060 | Seamount |
| 152 | United Nation Rise | Dublin | 33.49952 | 25.4763 | 2434 | Mud Volcano |
| 153 | United Nation Rise | Stoke-on Trent | 33.47922 | 25.55548 | 2551 | Mud Volcano |
| 154 | United Nation Rise | United Nation Rise | 33.59995 | 25.57977 | 2200 | Pockmarks |
| 155 | Ventotene Ridge | Ventotene | 40.7992 | 13.43237 | 100 | Volcanic Area |
| 156 | Western Mediterranean | Columbretes | 39.83604 | 0.680554 | 40 | Hydrothermal Vents |
