# Supplementary material for: Volcanic-associated ecosystems of the Mediterranean Sea: a systematic map and an interactive tool to support their conservation
Source: PeerJ. 2023 Mar 29;11:e15162. doi: 10.7717/peerj.15162 (PMC10066691; doi:10.7717/peerj.15162)
Supplement: Supplemental Information 5 — The number of observations, the percentage (on the total observation) and the average Depth Range (in m, minimum and maximum) were reported for the site types included in the Systematic Map. [file peerj-11-15162-s005.docx]

**Table S5.**

**Number of observations per Site Type**.

The number of observations, the percentage (on the total observation) and the average Depth Range (in m, minimum and maximum) were reported for the site types included in the Systematic Map.

| ***Site Type*** | **# of observations** | **% of observations by the total** | **Depth Range (min-max)** |
| --- | --- | --- | --- |
| Hydrothermal Vents | 473 | 47.9 | 1-3700 |
| Mud Volcano | 303 | 30.7 | 12-3030 |
| Cold Seeps | 57 | 5.8 | 12-2030 |
| Seamount | 54 | 5.5 | 497-3180 |
| Volcanic Area | 53 | 5.4 | 1-3180 |
| Pockmarks | 48 | 4.9 | 36-3800 |
