# Supplementary material for: Volcanic-associated ecosystems of the Mediterranean Sea: a systematic map and an interactive tool to support their conservation
Source: PeerJ. 2023 Mar 29;11:e15162. doi: 10.7717/peerj.15162 (PMC10066691; doi:10.7717/peerj.15162)
Supplement: Supplemental Information 6 — The code and status for Priority Habitat, Non-Priority Habitat and Species identified were reported based on the European Habitats Directive, SPA/BD Protocol, BERN Convention, IUCN Mediterranean, IUCN Italian and CITES lists. [file peerj-11-15162-s006.docx]

**Table S6:**

**List of protected habitat or species identified in the Aeolian Arc**.

The code and status for Priority Habitat, Non-Priority Habitat and Species identified were reported based on the European Habitats Directive, SPA/BD Protocol, BERN Convention, IUCN Mediterranean, IUCN Italian and CITES lists.

| Habitat/Species | European Habitats Directive | SPA/BD | BERN | IUCN Mediterranean | IUCN Italian | CITES |
| --- | --- | --- | --- | --- | --- | --- |
| Priority Habitat |  |  |  |  |  |  |
| *Posidonia oceanica* meadows | 1120 | III.5.1 |  |  |  |  |
| *facies* with *Eunicella singularis* | 1170 | IV.3.1.11 |  |  |  |  |
| *facies* with *Paramuricea clavate* | 1170 | IV.3.1.13 |  |  |  |  |
|  |  |  |  |  |  |  |
| Non-Priority Habitat |  |  |  |  |  |  |
| *Neopycnodonte* cochlear biogenic reef | 1170 |  |  |  |  |  |
| Submarine structures made by leaking gases | 1180 |  |  |  |  |  |
|  |  |  |  |  |  |  |
| Species |  |  |  |  |  |  |
| *Laminaria rodriguezii* |  | II |  |  |  |  |
| *Posidonia oceanica* |  | II | App. I |  |  |  |
| *Antipathella subpinnata* |  | II-III | App. 3 | NT-Near Threatened | LC-Least Concern |  |
| *Antiphates dichotoma* |  | II-III | App. 3 | NT-Near Threatened | LC-Least Concern | App. 2 C2 |
| *Corallium rubrum* |  | III | App. 3 |  |  |  |
| *Savalia savaglia* |  | II | App. 2 | E-Endangered | E-Endangered | App. 2 |
| *Dendrophyllia cornigera* |  |  |  | E-Endangered | V-Vulnerable | App. 2 |
| *Pinna nobilis* | IV | II | App. 2 | CE-Critically Endangered |  |  |
| *Palinurus elephas* |  | III | App. 3 |  |  |  |
| *Centrostephanus longispinus* | IV | II | App. 2 |  |  |  |
| *Ophidiaster ophidianus* |  | II | App. I |  |  |  |
