# Supplementary material for: Volcanic-associated ecosystems of the Mediterranean Sea: a systematic map and an interactive tool to support their conservation
Source: PeerJ. 2023 Mar 29;11:e15162. doi: 10.7717/peerj.15162 (PMC10066691; doi:10.7717/peerj.15162)
Supplement: Supplemental Information 7 — The flow-chart shows the stages of the screening process during the analysis of the literature included in the Systematic Map. Figure realized using the package metagear (Lajeunesse, 2016). [file peerj-11-15162-s007.docx]

**
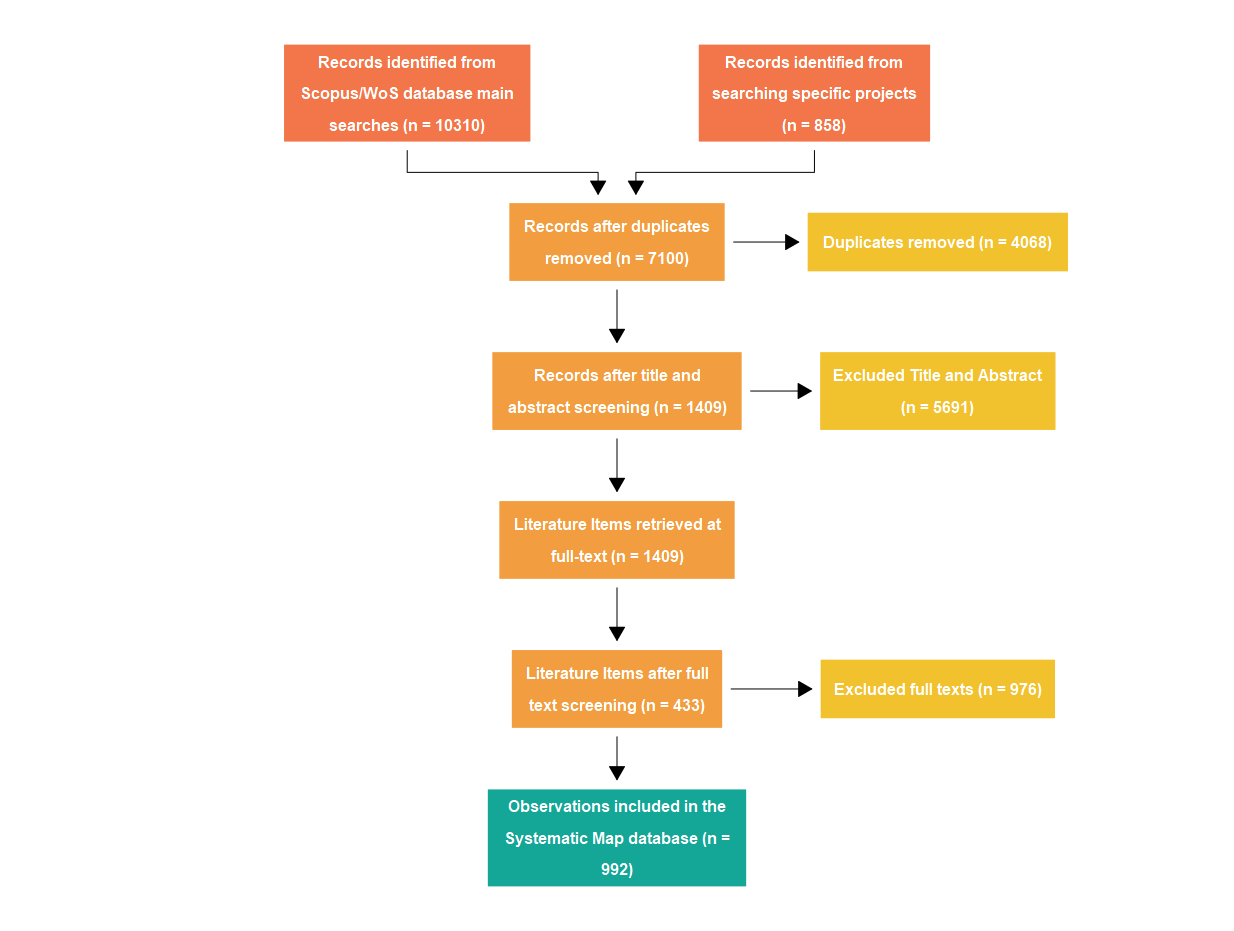
**

**Figure S1:** **ROSES flow chart**.

The flow-chart shows the stages of the screening process during the analysis of the literature included in the Systematic Map. Figure realized using the package *metagear* (Lajeunesse, 2016).
