# Supplementary material for: Volcanic-associated ecosystems of the Mediterranean Sea: a systematic map and an interactive tool to support their conservation
Source: PeerJ. 2023 Mar 29;11:e15162. doi: 10.7717/peerj.15162 (PMC10066691; doi:10.7717/peerj.15162)
Supplement: Supplemental Information 8 — The graphical output example of the “Interactive Map” section used the selection of all the Italian sites. [file peerj-11-15162-s008.docx]

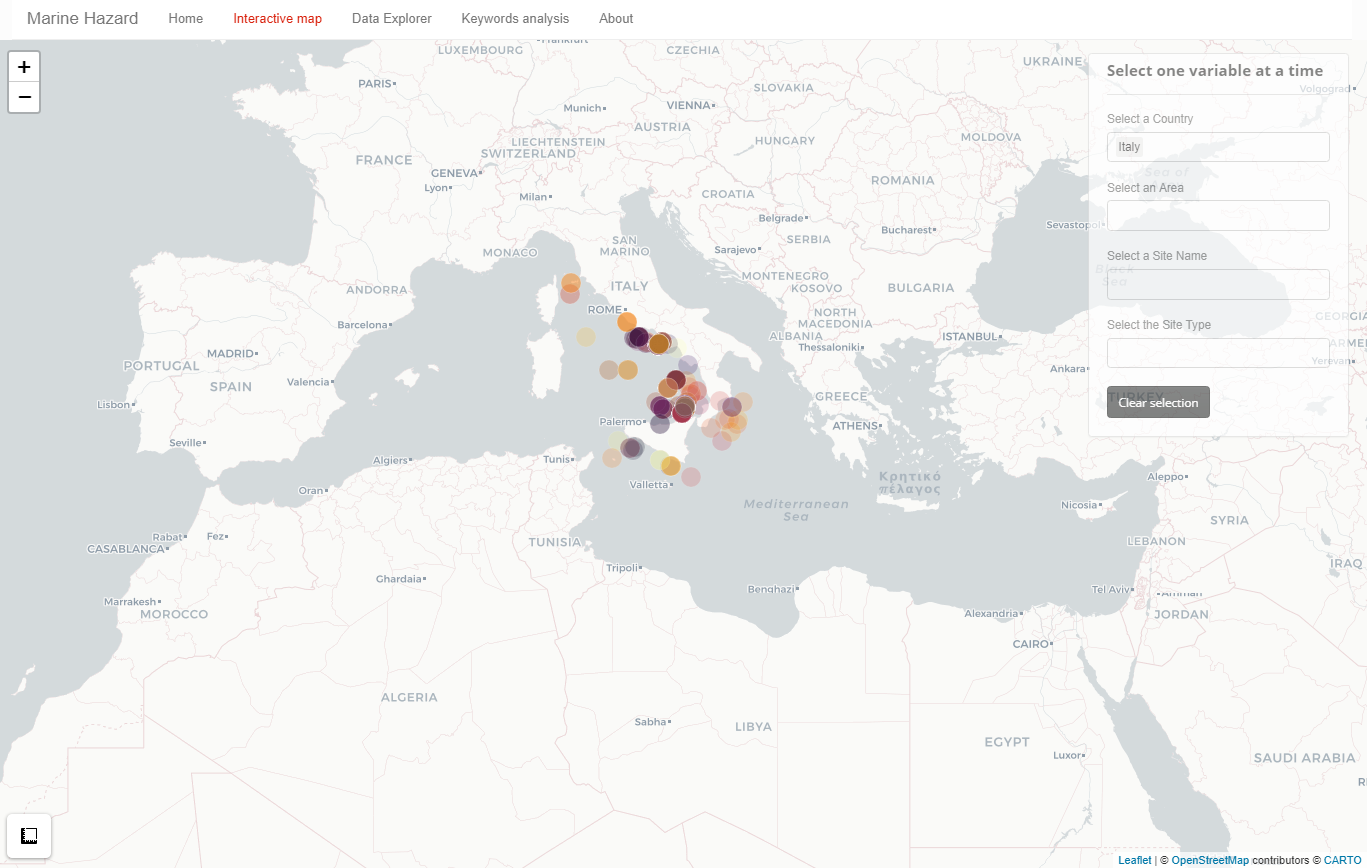


**Figure S2**: **Section “Interactive Map” of the *MH-shiny* app.**

The graphical output example of the “Interactive Map” section used the selection of all the Italian sites.
