# Supplementary material for: Volcanic-associated ecosystems of the Mediterranean Sea: a systematic map and an interactive tool to support their conservation
Source: PeerJ. 2023 Mar 29;11:e15162. doi: 10.7717/peerj.15162 (PMC10066691; doi:10.7717/peerj.15162)
Supplement: Supplemental Information 9 — The graphical output example of the “Data Explorer” section is based on the selection made in the “Interactive Map” section. [file peerj-11-15162-s009.docx]

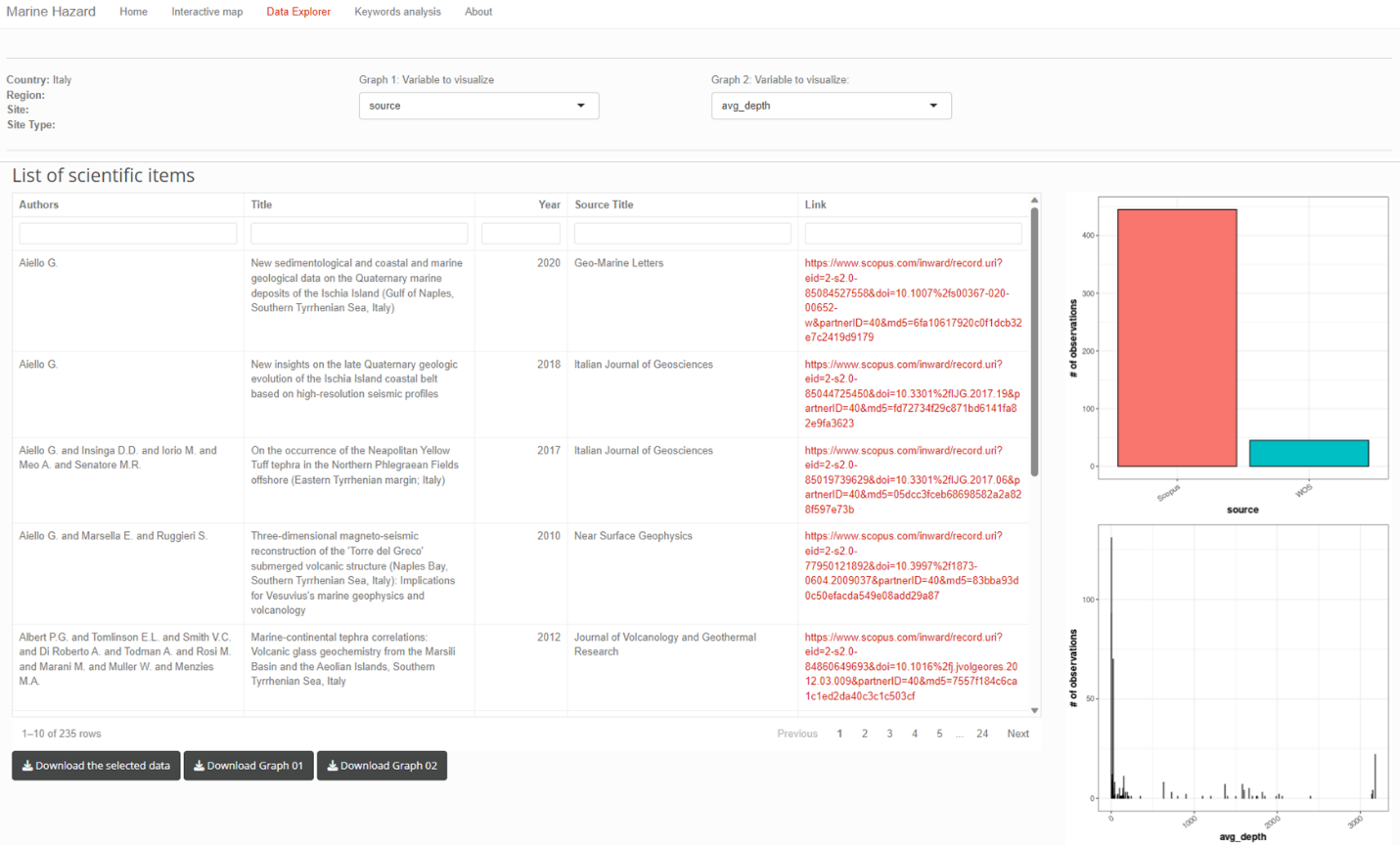


**Figure S3**: **Section “Data Explorer” of the *MH-shiny* app.**

The graphical output example of the “Data Explorer” section is based on the selection made in the “Interactive Map” section.
