# Supplementary material for: Volcanic-associated ecosystems of the Mediterranean Sea: a systematic map and an interactive tool to support their conservation
Source: PeerJ. 2023 Mar 29;11:e15162. doi: 10.7717/peerj.15162 (PMC10066691; doi:10.7717/peerj.15162)
Supplement: Supplemental Information 10 — The graphical output example of the “Keyword Analysis” section is produced with the selection of “author_keywords”, Minimum Frequency of 1 and Maximum Number of Words of 100 and Network analysis based on the selection made in the “Interactive Map” section. [file peerj-11-15162-s010.docx]

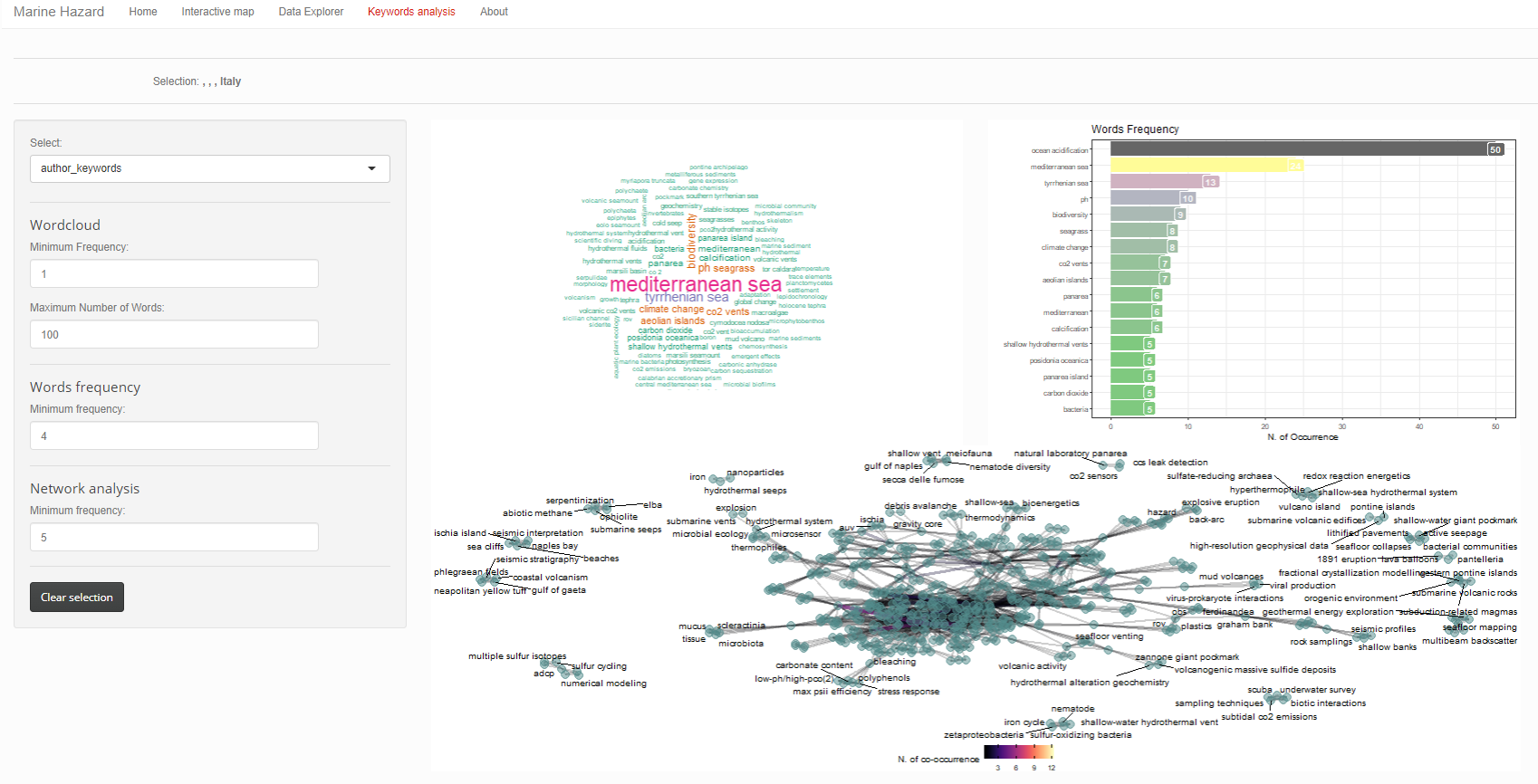


**Figure S4: Section “Keyword Analysis” of the MH-shiny app.**

The graphical output example of the “Keyword Analysis” section is produced with the selection of “author_keywords”, Minimum Frequency of 1 and Maximum Number of Words of 100 and Network analysis based on the selection made in the “Interactive Map” section.
